# BSEcho 2024 Conference Report

**DOI:** 10.1186/s44156-025-00079-y

**Published:** 2025-07-28

The annual conference is the BSE’s flagship event, and this year did not disappoint. Edinburgh was a fantastic host providing the perfect Scottish hospitality. The venue provided the required space and atmosphere to facilitate the world class presentations, debates, workshops and exhibitors. For those 580 delegates who attended face to face, the learning environment was complemented by the ability to network with colleagues and friends and for those additional 694 delegates who were able to join virtually, the recorded sessions provided the ideal solution for those who were unable to venture to the Scottish capital.

The conference was opened by Prof Daniel Augustine, our current president who created a positive and uplifting mood which was maintained across both days by dynamic speakers providing refreshing, novel and innovative content. There was an abundance of brilliance; integrated into well-planned sessions, providing something for everyone with themed content based on the epidemiology, the basics, the advanced and the future. The varied content was supported by two dedicated sessions developed in association with the British Cardio-Oncology Society and Advanced Echo Scotland. The final session of the conference provided an important but entertaining debate on the role of focussed echocardiography in the diagnosis and management of patients with heart failure. We were also delighted to welcome our international speaker, Prof Victoria Delgado who provided an amazing journey through the world of artificial intelligence in echocardiography and our invited speaker, Jane Lynch who gave an insightful account of the current and evolving world of Healthcare Science.

The parallel streams were complemented by a range of workshops. These were incredibly popular and provided delegates with the opportunity to interact, practise and learn from experts in the field. The content included diastolic function, prosthetic valves, quality assurance and aortic stenosis. We are extremely grateful to Ross Cullen (Olympic BMX GB rider) for giving up his time and his echo windows for the altruistic support of education during the sports cardiology workshop—a true athletes heart.

High quality research was a common thread throughout, embedded within presentations, highlighted in workshops and showcased on 26 poster abstracts. The Investigator of the Year Award was given the centre stage, and the investigators had the attention of the full auditorium to present their research findings. We would like to congratulate Christopher Wild for being a worthy winner and providing an excellent presentation of his study ‘*Echocardiographic assessment of pulmonary hypertension: novel markers to help identify pulmonary hypertension secondary to left heart disease’*.

This year we provided due recognition to 20 members who were awarded a fellowship of the BSE, truly acknowledging the amazing and often unseen efforts. In addition, it was a pleasure to be able to award two life-time achievement awards, to Prof Richard Steeds and Jane Lynch for their dedication and commitment to echocardiography and devoting a career that has had a lasting impact on every echocardiographer across the UK.

We are now preparing for Bournemouth 2025. We are extremely excited that this conference will build upon the success of Edinburgh 2024. In the meantime—keep reading, keep learning and enjoy your echocardiography.

**Professor David Oxborough and Dr Liam Ring**

Co-Chairs of Education

## ABS001: The value of evaluating cardiac damage in patients with aortic stenosis: A systematic review

### Sadie Bennett^1,2^, Eric Holroyd^1^, Maria F. Paton^3,4^, Paul Leeson^2,5^, Bjorn Redfors^6,7,8^, Philippe Pibarot^9^, Philippe Généreux^10^, Chun Shing Kwok^1^

#### ^1^University Hospitals of North Midlands, UK; ^2^Cardiovascular Clinical Research Facility, University of Oxford, UK; ^3^Leeds Teaching Hospitals NHS Trust, Leeds, UK; ^4^Faculty of Medicine and Health, University of Leeds, Leeds, UK; ^5^Oxford University Hospitals NHS Foundation Trust, John Radcliffe, Oxford, UK; ^6^Department of Molecular and Clinical Medicine, Gothenburg University, Gothenburg, Sweden; ^7^Department of Cardiology, Sahlgrenska University Hospital, Gothenburg, Sweden; ^8^Clinical trials Centre, Cardiovascular Research Foundation, New York City, USA; ^9^Laval University, Quebec City, Canada & Quebec Heart and Lung Institute, Quebec City, Canada; ^10^Gagnon Cardiovascular Institute, Morristown Medical Center, Morristown, USA

*Echo Research & Practice* 2025, **12(Suppl 1):**ABS001

**Abstract: Background:** Aortic stenosis (AS) is a common valvular heart disease. A novel scoring system based on ‘cardiac damage’ has been proposed recently that characterises pathophysiological consequences of AS into different stages. This staging system may be useful for assessment of risk and prognosis in patients with AS to guide treatment.

**Methods:** We conducted a systematic review of studies which evaluated ‘cardiac damage’ in patients with AS to assess its value in identifying high risk patients. A search of MEDLINE and EMBASE was performed in January 2024 with data being extracted from relevant studies. Studies were pooled numerically or in meta-analysis.

**Results:** A total of 18 studies were included with 21,876 patients (mean age 79 years, 52.7% males). For patients who underwent any AVR the pooled mortality for stage 0, 1, 2, 3 and 4 was 5.3% (6/114), 6.8% (22/325), 11.9% (118/995), 18.9% (92/486) and 21.6% (35/162), respectively. For patients with transcatheter AVR, the pooled mortality rate was 8.41% for stage 0 (96/1141), 17.08% for stage 1 (218/1276), 23.30% for stage 2 (624/2678), 32.46% for stage 3 and 36.47% for stage 4 (349/957). In comparison to stage 0, the odds risk (OR) of mortality for stage 1: OR 1.50 95%CI 1.14–1.98, stage 2: OR 1.77 95%CI 1.37–2.29, stage 3: OR 2.99 95%CI 2.28–3.94 and stage 4: OR 3.82 95%CI 2.68–5.44.

**Conclusions:** Assessment of ‘cardiac damage’ stage has strong prognostic value for patients with AS who require AVR.

## ABS003: An Evaluation of The Effect of Cardiac Resynchronisation Therapy on Myocardial Work Efficiency in Patients with Underlying Right Bundle Branch Block vs Left Bundle Branch Block

### Z. Rogers^1,2^

#### ^1^West Suffolk NHS Foundation Trust, Suffolk, UK; ^2^Manchester Metropolitan University, Manchester, UK

*Echo Research & Practice* 2025, **12(Suppl 1):**ABS003

**Background:** Myocardial work efficiency (MWE) is an emerging technique for the assessment of global and segmental left ventricular (LV) contraction. Studies thus far have demonstrated significant disparity between septal and lateral MWE in left bundle branch block (LBBB) patients which improves with cardiac resynchronisation therapy (CRT). There is a lack of research measuring MWE in right bundle branch block (RBBB) patients.

**Purpose:** To compare the effect of CRT on MWE in patients with underlying RBBB versus LBBB. To assess differences in baseline MWE patterns.

**Methods:** Data was retrospectively collected from July 2020 until July 2023. Two groups of 10 patients were compared (Group 1: LBBB, Group 2: RBBB). MWE analysis was performed on pre-implant and post-implant echocardiograms. The effect of CRT on global and regional MWE was evaluated and correlations between the change in Global Work Efficiency (GWE) and other indicators of LV function were assessed.

**Results:** Overall group 1 showed high baseline lateral MWE and low septal MWE (Fig. 1). Group 2 showed no consistent baseline MWE pattern. Baseline GWE was lower in group 1 and significantly increased post-CRT (*p* = 0.001). GWE did not significantly change in group 2 (*p* = 0.37). Increases in GWE correlated with significant reductions in LV volumes in group 1 and baseline septal WE was strongly associated with the degree of reduction in LV volumes (*p* = 0.015 and *p* = 0.018).

**Fig. 1 (abstract ABS003):**
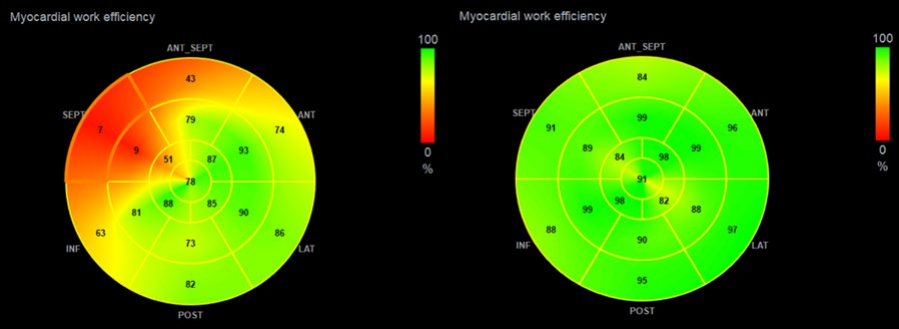
Example MWE Pattern pre and post-CRT in a LBBB Patient

**Conclusions:** MWE may be a valuable tool for identifying patients with significant mechanical dyssynchrony which acts as a substrate for improvement via CRT. Further research with greater sample sizes is needed to assess whether measurement of baseline MWE could predict response to CRT particularly in RBBB patients.

## ABS004: Effect of home-based physical activity intervention on cardiac structure and function in middle-aged and older otherwise healthy individuals with a history of COVID-19

### Mushidur Rahman^1,2^, Sophie L. Russell ^1,2^, Nduka C. Okwose ^1,2^, Olivia M. A. Hood^3^, Helen Maddock ^1^, Prithwish Banerjee^1,2^,, Djordje G. Jakovljevic^1,2^

#### ^1^Clinical Sciences and Translational Medicine Theme, Research Centre for Health and Life Sciences, Coventry University, Coventry, UK; ^2^Department of Cardiology, University Hospitals Coventry and Warwickshire NHS Trust, Coventry, UK; ^3^School of Biosciences, College of Biomedical and Life Sciences, Cardiff University, UK

*Echo Research & Practice* 2025, **12(Suppl 1):**ABS004

**Background:** Coronavirus disease 2019 (COVID-19) has been reported to cause cardiac functional and structural remodelling. Physical activity is known to improve cardiovascular function and reduce cardiac related hospitalisation.

**Purpose:** To evaluate the effects of a home-based physical activity intervention on cardiac structure and function in healthy middle-aged and older individuals with a history of COVID-19.

**Methods:** A single centre, randomised controlled study enrolled eighty-four individuals with a history of COVID-19. Participants were free of underlying cardiac and or respiratory conditions. Cardiac structure and function were assessed using echocardiography at rest and during peak-exercise and heart rate variability (HRV) at rest. Participants were randomly assigned (1:1) to either a 12-week home-based physical activity group (n = 42) or usual care control group (n = 42). The control group were asked to continue with usual physical activities whilst the intervention group were asked to increase their number of steps by 2000 steps per-day from baseline (Active-at-Home).

**Results:** Average step-count increased from baseline to follow-up by 1897 steps/day in the intervention group (baseline: 7377 ± 3821 steps/day; follow-up: 9274 ± 5337 steps/day, respectively, *p*-value = 0.001). No significant difference was seen in left ventricular mass index and relative wall thickness between control and intervention groups [*p*-value > 0.05]. However, a significant between group difference was seen in left ventricular mass to volume ratio [95% CI: 0.20–0.36, *p*-value = 0.032]. No significant between group difference was detected in measures of HRV.

**Conclusion:** Home-based physical activity intervention has a limited effect on cardiac structure and function in middle-aged and older otherwise healthy individuals with a history of COVID-19. The present study shows a home-based physical activity intervention can promote an active lifestyle safely by increasing daily activity levels.

## ABS005: Dedicated transthoracic echocardiography in acute medicine

### Joseph Bradley

#### University Hospitals Birmingham NHS Foundation Trust, UK

*Echo Research & Practice* 2025, **12(Suppl 1):**ABS005

**Introduction/aims**: This service improvement project aimed to assess the impact of a dedicated Acute Medicine Cardiology Diagnostic Lead (AMCDL), a band 8 cardiac physiologist screening/performing cardiac diagnostic tests, on the utilisation and outcomes of patients requiring Transthoracic Echocardiography (TTE) and 24-h ambulatory ECG (AECG). For the purpose of BSE, only TTE was analysed.

**Method**: We performed an audit of all patients with requests for TTE, from Acute Medicine at Queen Elizabeth Hospital, Birmingham, for the first 8 months of the AMCDL role implementation (September 2023-April 2024, n27683) and compared these to the same period of the previous year (September 2022-April 2023, n2525). Time from patient admission to discharge, time from request to completion and location that TTE was performed was analysed.

**Results:** There was a noticeable reduction in average waiting time from request to completion of TTE, with an average reduction of 3.2 days for inpatients and 3.4 days for outpatients.

The average inpatient stay considerably reduced by 0.5 days, average 346 patients per month equals 173 bed days saved per month. With the cost of an inpatient bed at the QE of £215, this equates to average savings of £37,195 per month.

The number of TTE’s performed in Acute Medicine greatly improved from 25 to 45%.

**Conclusions**: Preliminary data shows that targeted, triaged early access to TTE improves patient wait times and reduces length of stay, providing significant cost saving. The current positive results were achieved with a single staff member as the AMCDL, a business case has since been approved to employ more cardiology diagnostic staff assigned to Acute Medicine to further improve results. Initial analysis highlights that a number of TTE’s provided significant early diagnosis and/or early discharge; additional work will be done to confirm full impact on patient pathways.

## ABS006: Echocardiography in Duchenne muscular dystrophy: A call for consistency and standardisation

### Lynne Williams^1^, Sadie Bennett^2^, Charlotte Atkinson^3^, Daniel X. Augustine^4^, Maria Bland^5^, Hatty Grant^6^, Jade Hobday^7^, Anna Johnson^5^, Kadhim Kadhim^5^, Lisa Kuhwald^8^, Chiara Marini Bettolo^5^, David Oxborough^9^, Liam Ring^10^, Shaun Robinson^11^, Jo Sopala^6^, Chet Villa^12^, Michela Guglieri^13^, John Bourke^5^, Caroline Coats^14^

#### ^1^Royal Papworth Hospital NHS Foundation Trust, UK; ^2^University Hospitals of North Midlands NHS Trust, UK; ^3^University Hospital Southampton NHS Trust, UK; ^4^Royal United Hospitals Bath NHS Trust, UK; ^5^Newcastle upon Tyne Hospitals NHS Foundation Trust, UK; ^6^British Society of Echocardiography, UK; ^7^Birmingham Women's and Children’s NHS Trust, UK; ^8^Duchenne UK; ^9^Liverpool John Moore's University, UK; ^10^West Suffolk NHS Foundation Trust, UK; ^11^Imperial College Healthcare NHS Trust, UK; ^12^Cincinnati Children’s Hospital Medical Center, USA; ^13^Newcastle University; ^14^University of Glasgow, UK

^*^Presenting author

*Echo Research & Practice* 2025, **12(Suppl 1):**ABS006

**Background:** Duchenne muscular dystrophy (DMD) is an inherited muscle-wasting disease caused by lack of dystrophin, essential to muscle integrity. Cardiomyopathy is inevitable and contributes to premature death (UK life expectancy 29 years). Conventional heart failure medications slow the decline of left ventricular (LV) systolic function, particularly when deployed prophylactically.

**Purpose:** The DMD Care UK cardiac working group published guidelines recommending empiric use of heart medications, a schedule of cardiac imaging and subsequent application of results in clinical decision-making.

Accurate, reproducible echocardiographic measurements are crucial in monitoring LV-function. However, there is considerable inter-departmental variation in the acquired measurements and generated report and differences between paediatric vs adult centres. This reduces test-value and contributes to spurious variations in measures over time. A UK-wide DMD standardised echocardiography framework will address this.

**Methods:** A specialised echocardiography imaging protocol will be developed (by early 2025) through review, consultation, consensus building, endorsement and publication. This process will be coordinated by the authors as part of DMD Care UK.

**Results:** Themes that have been identified to be addressed include:

- Can echocardiography be better standardised, allowing amalgamation of results over time (eg: local hospital vs specialist centre)?
- Which echocardiography measurements can be obtained reliably at every assessment, particularly when patients’ mobility worsens and echocardiography imaging windows become limited?
- Could a composite measure, including LV-strain and LV volumes be more clinically meaningful than LV ejection fraction alone?
- Should measures of regional LV function be routinely reported (eg: wall motion scoring; tissue-Doppler measures; speckle tracking)?
- When echocardiography imaging windows limit accurate LV function assessment, should LV opacification studies be undertaken routinely?

**Conclusion:** We seek to standardise echocardiography protocols to improve clinical decision-making in DMD. Furthermore, this would allow for improved echocardiography endpoints for DMD research studies.

## ABS007: The feasibility, reproducibility and accuracy of three-dimensional echocardiography in diagnosing cardiac sarcoidosis: a prospective study

### Joseph Okafor^1,2,4^, Alessia Azzu^2,3^, Raheel Ahmed^2,4^, Athol Wells^4^, A. John Baksi^3,4^, Vasileios Kouranos^4^, Kshama Wechalekar^4,5^, Roxy Senior^1,2^, Peter Collins^2^, Rakesh Sharma^4^, Rajdeep Khattar^1,2,4^

#### ^1^Department of Echocardiography, Royal Brompton & Harefield Hospitals, Guy’s and St. Thomas’ NHS Foundation Trust, London, United Kingdom; ^2^National Heart & Lung Institute, Imperial College London, United Kingdom; ^3^Cardiovascular Magnetic Resonance Unit, Royal Brompton & Harefield Hospitals, Guy’s and St. Thomas’ NHS Foundation Trust, London, United Kingdom; ^4^Cardiac Sarcoidosis Service, Royal Brompton & Harefield Hospitals, Guy’s and St. Thomas’ NHS Foundation Trust, London, United Kingdom; ^5^Department of Nuclear Medicine and PET, Royal Brompton & Harefield Hospitals, Guy’s and St. Thomas’ NHS Foundation Trust, London, United Kingdom

*Echo Research & Practice* 2025, **12(Suppl 1):**ABS007

**Introduction:** Three-dimensional echocardiography (3DE) offers several technical advantages over two-dimensional imaging including less geometrical assumptions. The feasibility, reproducibility and diagnostic ability of 3DE in cardiac sarcoidosis (CS) has yet to be explored.

**Methods:** Consecutive patients referred for evaluation of suspected CS were prospectively recruited and underwent transthoracic echocardiography with 3D imaging and speckle-tracking, cardiac magnetic resonance (CMR) and ^18^F-FDG-PET. The diagnosis of CS was made using the Heart Rhythm Society criteria. Subgroup analysis among all patients in whom 3DE was feasible was performed.

**Results:** Of the 240 patients recruited (age 55 ± 11 years, 60% male), 3DE was feasible in 106 (44%) and 46 (43%) of these had CS. CS patients had lower 3DE LVEF (57% [49–61] vs 59% [55–62], p = 0.034), RVEF (48% [44–51] vs 52% [46–57], p = 0.024) and torsion (1.2°/cm [0.9–1.6] vs 1.6°/cm [0.9–2.4], p = 0.019). There was no significant difference in global longitudinal strain, circumferential strain, radial strain, principal strain and twist between the two groups. Intra-reader reliability assessed by intraclass correlation coefficient (ICC) was greatest for global longitudinal strain (ICC 0.763) and lowest for global radial strain (ICC 0.569).

Among the 3DE parameters, LVEF (AUC 0.63) with an optimal cut-off of < 50% (sensitivity 28%, specificity 91%) and torsion (AUC 0.63) with cut-off < 2.15°/cm (sensitivity 90%, specificity 38%) had the best ability to detect CS. The AUC for ^18^F-FDG-PET and CMR was 0.68 and 0.88, respectively.

**Conclusion:** Three-dimensional echocardiography has modest feasibility and reproducibility in CS patients but when available, torsion may provide incremental diagnostic value over two-dimensional imaging.

**Table 1 (abstract ABS007):** Left ventricular strain parameters assessed using 3D speckle-tracking echocardiography among cardiac sarcoidosis (CS) patients and inter-reader reliability

Strain parameter	Whole group (n=106)	CS+ (n=46)	CS- (n=60)	P-value	ICC*	95% CI
GLS, % (± SD)	− 19.4 ± 5.6	− 18.4 ± 6.8	− 20.2 ± 4.6	0.097	0.763	0.559–0.879
GCS, % (± SD)	− 27.4 ± 6.7	− 26.8 ± 6.6	− 28.2 ± 6.6	0.240	0.746	0.532–0.871
GRS, % (± SD)	30.7 ± 11.5	35.8 ± 14.8	40.7 ± 7.7	0.134	0.569	0.269–0.769
GPS, % (± SD)	− 33.1 ± 7.3	− 32.5 ± 7.8	− 33.9 ± 6.8	0.303	0.650	0.383–0.816
Twist, % (IQR)	11.9 (7.6–17.8)	10.8 (7.9–14.4)	12.9 (7.5–20.1)	0.126	0.718	0.486–0.854
Torsion°/cm (IQR)	1.4 (0.9–2.1)	1.2 (0.9–1.6)	1.6 (0.9− 2.4)	0.019	0.701	0.461–0.848

*Intraclass correlations (ICC) assessed among 30 repeated studies using two-way mixed-effects model. GCS = global circumferential strain; GLS = global longitudinal strain; GPS = global principal strain; GRS = global radial strain

## ABS008: Left ventricular systolic function assessment in patients with atrial fibrillation and rapid ventricular response

### Simon Smith, Sekai Sengwe, Steven Hodgson, Matthew Dewhurst, Helen Oxenham

#### North Tees and Hartlepool NHS Foundation Trust, United Kingdom

*Echo Research & Practice* 2025, **12(Suppl 1):**ABS008

**Background**: Echocardiography is often not performed in patients with AF and rapid ventricular response (RVR) because systolic function assessment in this context is assumed to be inaccurate. Heart failure commonly co-exists with AF and identification of left ventricular systolic impairment (LVSI) is important for early initiation of appropriate therapy.

**Methods**: We retrospectively analysed Echocardiogram reports of patients with heart rates (HR) > 100bpm over a 3-month period in a single NHS Trust. Left Ventricular (LV) function was categorised using British Society of Echocardiography reference ranges. Repeat echo data for 57% of patients were also analysed.

**Results**: 69 patients had HR > 100bpm and LV ejection fraction could be assessed in 100% of cases. 72% of patients had AF and were more likely to have impaired (58% vs. 16%, p < 0.05) or severely impaired (34% vs. 16%, p < 0.05) LV function compared to patients in sinus tachycardia. 17 (57%) patients had repeat echocardiography 3 months after initiation of at least 2 pillars of heart failure therapy, of which, 11 (65%) had persistent LVSI, 3 (17%) of which remained severely impaired.

**Conclusions and recommendations**: LV function can be estimated in patients with AF and RVR. The incidence and severity of LVSI was greater than patients in sinus tachycardia. Whilst some patients showed improvement, most had persisting LVSI despite adequate rate control. Protocols that limit access to diagnostic echo in patients with AF and RVR could delay identification and management of patients with co-existing LV systolic dysfunction.

**Table 1 (abstract ABS008):** Initial reported LV function and cohort demographics

LV function	AF (n = 50)	Sinus (n = 19)
Normal	17 (34%)	13 (68%)
Borderline low	4 (8%)	1 (5%)
Impaired	12 (24%)	2 (11%)
Severely impaired	17 (34%)	3 (16%)
HR (bpm), mean (SD)	116 (18)	114 (16)
Male	28	7
Female	22	12
Age (years), mean (SD)	75 (10.3)	55 (17.9)
BMI (kg/m^2^), mean (SD)	30.6 (7.5)	28.6 (7.8)

**Fig. 2 (abstract ABS008):**
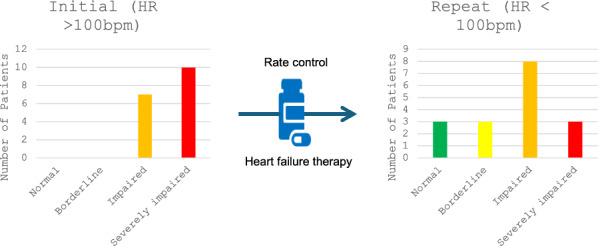
LV function in patients who had repeat Echocardiography

## ABS009: Appropriate use of ultrasound enhancing agent echocardiography in the assessment of left ventricular function. A trust-wide service evaluation and improvement study

### Tom Curran, Jennifer Vickers

#### University Hospitals Birmingham (UHB) NHS Foundation Trust, UK

*Echo Research & Practice* 2025, **12(Suppl 1):**ABS009

**Background:** Evaluation of left ventricular ejection fraction (LVEF) by transthoracic echocardiography (TTE) is a cornerstone of cardiac diagnostics. However, approximately 20% of TTE’s result in poor endocardial border definition^(1,2)^, reducing accuracy of LVEF assessments. Ultrasound enhancing agents (UEA) can be introduced to enhance endocardial border delineation and improve accuracy of LVEF measurements. *American Society of Echocardiography* (ASE) guidelines indicate UEA for LVEF quantification whenever images are suboptimal, nevertheless, UEA’s are widely underutilised.

**Purpose:** To appraise *UHB NHS Foundation Trust’s* compliance to ASE guidelines for the use of UEA in the assessment of LV function and assess improvement following an education program.

**Methods:** A two-phase retrospective audit design was used to evaluate 200 TTE’s indicated for assessment of LV function, across 4 hospital sites. TTE’s were reviewed by 2 BSE accredited echocardiographers to determine if studies met ASE echocardiographic criteria for UEA, if this was correctly identified and were subsequently referred for UEA. Following education, a further 200 TTE’s were analysed using the same protocol, with improvement assessed by chi-squared test (95% CI, P = 0.05) or Fischer’s exact test (95% CI, p = 0.05). A questionnaire was distributed to gain qualitative information regarding UEA services.

**Results:** Trust referral rates for UEA were substantially lower than those meeting criteria in both phases (13.6% and 14.2% respectively) (Fig. 1 and Fig. 2). No significant improvement was demonstrated following education program: χ^2^(1,n = 149) = 0.110,p = 0.741. A need for doctors to be present for UEA was identified as the biggest barrier to performance within the trust (Fig. 3).

**Conclusions:** Results suggest non-compliance to guidelines and underutilisation of UEA; however, the small sample size may be misrepresentative. Expanding physiologist-led UEA services may alleviate various barriers to UEA performance including medical supervision, time constraints and specialist training, as well as improve compliance to guidelines. Larger studies should therefore be conducted with a view to implement meaningful changes to service provisions. The study may be a useful pilot for other trusts to evaluate their own use of UEA.

## References

1. Crouse LJ, Cheirif J, Hanly DE, Kisslo JA, Labovitz AJ, Raichlen JS, et al. Opacification and border delineation improvement in patients with suboptimal endocardial border definition in routine echocardiography: Results of the phase III albunex multicenter trial. J Am Coll Cardiol. 1993 Nov 1;22(5):1494–500.
2. Kornbluth M, Liang DH, Paloma A, Schnittger I. Native Tissue Harmonic Imaging Improves Endocardial Border Definition and Visualization of Cardiac Structures. J Am Soc Echocardiogr. 1998 Jul 1;11(7):693–70

**Fig. 1 (abstract ABS009):**
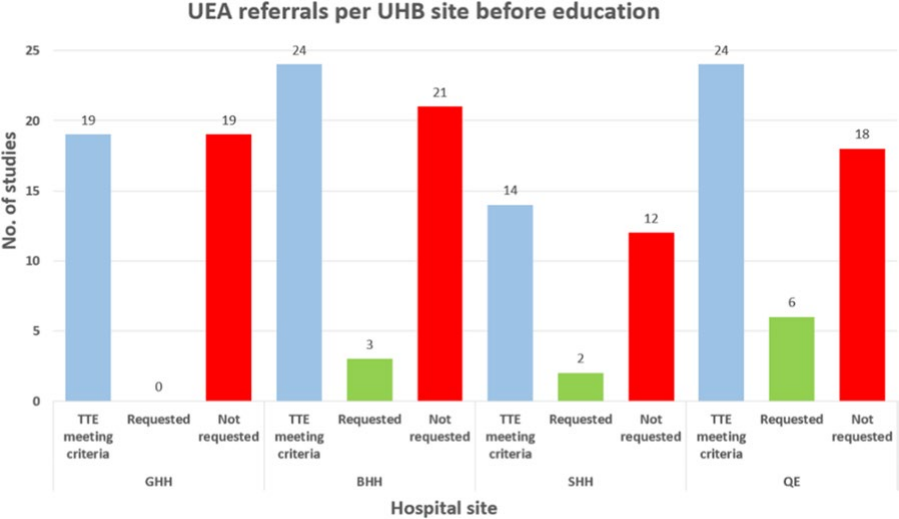
The number of TTE studies meeting criteria for UEA study and the number of UEA’s requested per hospital site from the 200 TTE’s selected in phase one

**Fig. 2 (abstract ABS009):**
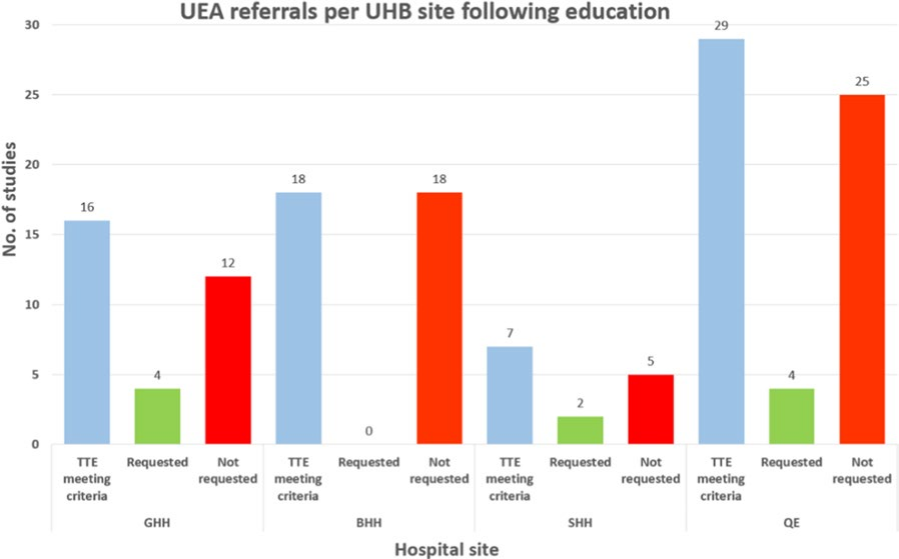
The number of TTE studies meeting criteria for UEA study and the number of UEA’s requested per hospital site from the 200 TTE’s selected in phase two

**Fig. 3 (abstract ABS009):**
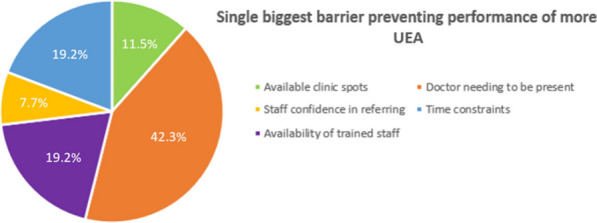
Staff feedback on the single biggest barrier to performing more LVO studies within the trust

## ABS010: Evaluation of patient satisfaction seen within Clinical Scientist led Echocardiography Clinics

### Jane Draper, Dario Freitas, Can Zhou. (joint first authors), Masha Sivalinganathan, Mitchell Fenn, Nathalie Aure, Anna Hayes

#### Cardiology Department, Guy’s and St Thomas’ NHS Foundations trust, London, UK

*Echo Research & Practice* 2025, **12(Suppl 1):**ABS010

**Background/introduction**: Demand on Consultants services and long waiting list have driven new ways of working within the NHS. Clinical Scientist (CS) led Echocardiography clinics are one such innovation providing care for specific patient cohorts. However, patient satisfaction within these services is poorly documented.

**Purpose**: To evaluate patient satisfaction of care within CS lead Echocardiography clinics within Adult Congenital Disease (ACHD), Valve, Cardio-Oncology (CO) and Inherited conditions (ICC) specialities.

**Methods**: 20 consecutive patients within each specialty clinics completed a locally developed satisfaction questionnaire at the end of consultations. This was completed anonymously on paper with sealed envelope return. Patients were asked to grade their level of agreement across specific areas of the consultation.

**Results**: Surveys were collected December 2023 to April 2024. A total of 80 patients participated, 20 from each specialty. 27% of patients were new, the remainder were follow-ups. Across each speciality all patients agreed or strongly agreed that they were treated in a courteous manner, that the CS had appropriate specialist knowledge and provided clear results and follow up plans. 88% of patients strongly agreed, that they had confidence in the advice they were given and that they were satisfied with their care. There was no difference in levels of satisfaction across specialty clinics or between whether the patient was new to the service or a follow up.

**Conclusions**: Overall patients agree or strongly agree that they are confident in the knowledge and review provided in CS clinics and are satisfied with the care they received. Future surveys may also be considered to ensure continuous improvement, but these findings support the implementation and potential expansion of CS-led echocardiography services within the NHS.

## ABS011: Appraising the performance of an echo department in a rural practice

### Gopinadh Pyla, Kyle Mooteeram, Paula Sheerin, Monica Monaghan, Eveline Lee

#### Department of Cardiology, South West Acute Hospital, Western Health and Social Care Trust, Enniskillen, UK

*Echo Research & Practice* 2025, **12(Suppl 1):**ABS011

**Background:** Echocardiography is essential in the diagnosis and management of cardiology issues. Our Trust serves a rural population of 300,000 across a vast area. There are 3 Echo departments providing independent assessments. This study aims to evaluate consistency of our echo service.

**Purpose:** To determine the repeatability of our echocardiographic measurements.

**Methods:** Over 12 weeks, all echo practitioners took part in categorical grading of mitral regurgitation, aortic stenosis, left and right ventricular function, and diastolic function. Numerical measurements included are PISA radius, LVOT diameter, AV Vmax, AV VTI, and LVOT VTI. Categorical data was analysed using Fleiss' Kappa and numerical data with Coefficient of Variation for assessment of inter- and intra-observer variability. Fleiss’ Kappa value of 1.00 denotes perfect agreement. CoV value of 0 denotes no variability in measurements.

**Results:** Interobserver variability showed an average Fleiss' Kappa of 0.74 (MR 0.56, AS 0.56, LVF 0.56 & RV function 1.0, diastolic function 1.0). Average CoV 7.44% (PISA radius 11.39%, LVOT diameter 8.5%, AV Vmax 3.74%, AV VTI 8.9%, LVOT VTI 4.69%).

Intraobserver variability showed an average Fleiss' Kappa of 0.82 (MR 1.0, AS 0.54, LVF 0.55, RV function 1.0, diastolic function 1.0) and an average CoV of 4.77% ( PISA 7.21%, LVOT diameter 5.29%, AV Vmax 2.50%, AV VTI 6.97%, LVOT VTI 1.89%).

**Fig. 1 (abstract ABS011):**
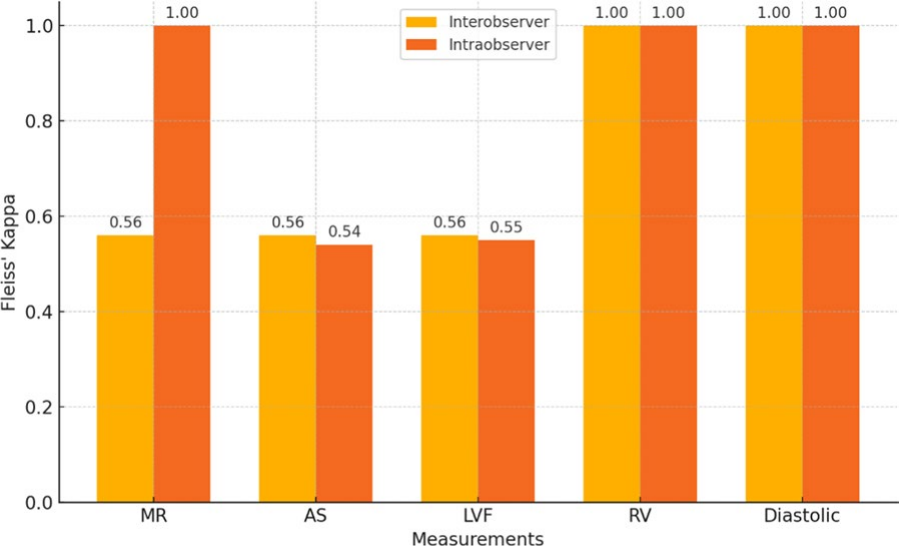
Comparison of Fleiss’ Kappa for Interobserver and Intraobserver Variability

**Fig. 2 (abstract ABS011):**
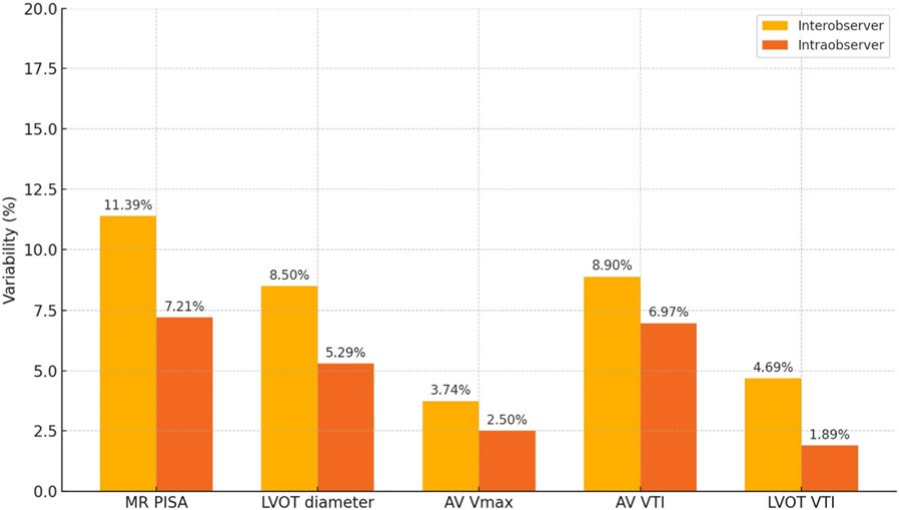
Interobserver and Intraobserver Variability

**Conclusions:** Our departmental results are consistent with published data. Test repeatability is, expectedly, less variable within observer than between observers. These findings support our readiness in achieving departmental accreditation.

## ABS012: Echocardiographic Assessment Of Pulmonary Hypertension: Novel Markers To Help Identify Pulmonary Hypertension Secondary To Left Heart Disease

### Christopher James Basil Wild^1,2^, Joseph Page^1^, Anil Gurung^1^, James Willis^1^, Oliver Slegg^1^, Jennifer Rossdale^1^, Jay Suntharalingham^1^, Robert Mackenzie Ross^1^, Daniel Knight^3^, A Hameed^4^, Athanasios Charalampopoulos^4^, David Oxborough^6^, Martin Johnson^5^, Dylan Thompson^2^, Oliver Peacock^2^, Nina Karia^3^, Gerry Coghlan^3^, Daniel X Augustine^1,2^

#### ^1^Royal United Hospitals Bath NHS Foundation Trust, Bath, UK. ^2^Department for Health, University of Bath, Bath, UK. ^3^Royal Free Hospital NHS Foundation Trust, London, UK. ^4^Sheffield Teaching Hospitals NHS Foundation Trust, Sheffield, UK. ^5^Golden Jubilee Hospital, Glasgow, UK. ^6^Liverpool John Moores University, Liverpool, UK

*Echo Research & Practice* 2025, **12(Suppl 1):**ABS012

**Background:** Pulmonary hypertension (PH) secondary to left heart disease is distinguished by a pulmonary capillary wedge pressure (PCWP) > 15 mmHg at right heart catheter (RHC). Inouke et al. (2024) propose a transthoracic echocardiogram (TTE) algorithm to discriminate pre- and post-capillary PH. We evaluated the potential of this algorithm in well characterised PH population, alongside certain diastolic TTE parameters, to improve the detection of post-capillary PH and/or generate an alternative algorithm.

**Methods:** Retrospective analysis of all patients referred for initial PH assessment. All patients underwent transthoracic echocardiogram (TTE) and gold standard RHC (38 ± 49 days). TTE measurements were evaluated by a single operator using Phillips Tomtec Arena 2020. T-test, receiver operator characteristic curve and crosstabulation was used for statistical analysis.

**Results:** 402 patients were included (63% female). 26% (n = 103) had PCWP > 15 mmHg at RHC. (Mean differences between TTE parameters are shown in Table 1) The proposed algorithm for detecting post-capillary PH yielded a sensitivity and specificity of 31% and 89% respectively. LV Global Longitudinal Strain was unable to differentiate pre- and post-capillary PH (p = 0.127). Significant differences were seen in indexed LA volume, reservoir and contractile strain, and mitral E/e’ (p < 0.001).

Analysing patients who had a full complement of TTE variables, a scoring system with a total of ≥ 2 points from any combination of three variables (Fig. 1) produced a sensitivity 85% and specificity 68% for the detection of post-capillary PH respectively.

**Conclusions:** An alternative algorithm using a combination of indexed LA volume, E/e’ and LA reservoir strain can discriminate pre- and post-capillary PH with good sensitivity and acceptable specificity in this select population. Further prospective studies are needed to assess the real-world application of this algorithm.

**Table 1 (abstract ABS012):** Statistical significance and Receiver Operator Characteristic curve-derived optimal cut-off values of novel left and right heart values in the assessment of pre- and post-capillary PH as determined by gold standard RHC

TTE parameter	PCWP ≤15mmHg (95% C.I)	PCWP >15mmHg (95% C.I)	Statistical significance	AUC	Optimum cut-off (Sns%/Spc%)
LA volume (ml)	48 ± 24	75 ± 33	< 0.001	0.77	47 (0.80/0.60)
Indexed LA volume (ml/m^2^)	26 ± 15	38 ± 18	< 0.001	0.73	28 (0.71/0.68)
RA area (cm^2^)	19 ± 8	24 ± 9	< 0.001	0.66	19 (0.70/0.52)
Indexed RA area (cm^2^/m^2^)	10 ± 4	12 ± 5	0.006	0.62	
LAsR (%)	24 ± 10	18 ± 10	< 0.001	0.69	21 (0.70/0.40)
LAsCd (%)	− 11 ± 8	− 10 ± 5	0.570		
LAsCt (%)	− 13 ± 7	− 8 ± 8	< 0.001	0.69	− 11 (0.70/0.61)
LVEF (%)	57 ± 7	57 ± 10	0.467		
LV GLS (%)	− 17 ± 3	− 13 ± 13	0.127		
Mean E/e’	7 ± 4	10.8 ± 7.9	< 0.001	0.66	8.2 (0.65/0.64)
MV E (cm/s)	0.5 ± 0.2	0.9 ± 0.3	< 0.001	0.75	0.62 (0.70/0.63)
MV E:A	0.8 ± 0.5	1.1 ± 0.7	0.006	0.61	
RAsR (%)	24 ± 13	18 ± 12	0.006	0.61	
RAsCd (%)	− 11 ± 7	− 8 ± 9	0.005	0.58	
RAsCt (%)	− 14 ± 9	− 10 ± 9	0.012	0.61	

LA: Left atrium, RA: Right atrium, LAsR: Left atrial reservoir strain, LAsCd: Left atrial conduit strain, LAsCt: Left atrial contractile strain, LVEF: Left ventricular ejection fraction, LV GLS: Left ventricular Global Longitudinal Strain, RAsR: Right atrial reservoir strain, RAsCd: Right atrial conduit strain, RAsCt: Right atrial contractile strain

**Fig. 3 (abstract ABS012):**
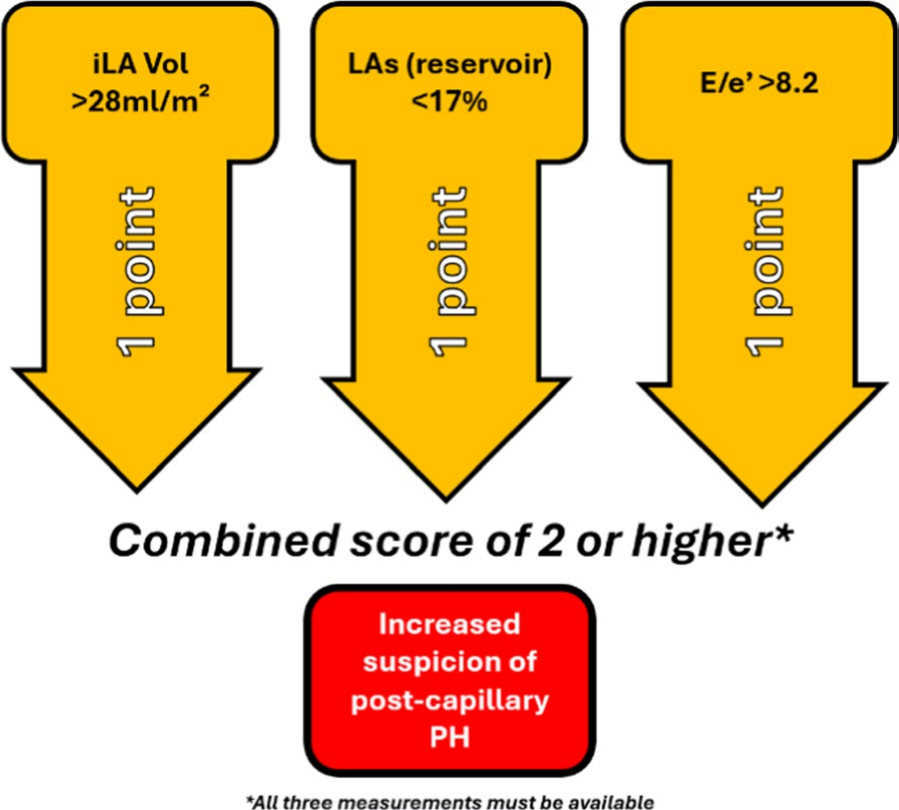
Scoring system for increased post-capillary PH suspicion, using a combination of any two commonly-acquired TTE measurements; indexed left atrial volume (iLA Vol), left atrial reservoir strain (LAs reservoir), and mitral E/e'

## ABS013: Piloting a physiologist led heart failure assessment clinic for non-urgent referrals

### Jonathan Cook, Jin Jiang, Ayisha Khan-Kheil, Dr Thomas Ingram

#### Royal Wolverhampton NHS Trust, UK

*Echo Research & Practice* 2025, **12(Suppl 1):**ABS013

**Background:** NICE guidelines state that patients with suspected heart failure (HF) and an NT-proBNP between 400–2000 ng/l, should have specialist assessment & transthoracic echocardiography within 6 weeks.

In line with NHS workforce plans to utilise and develop non-medical roles a pilot Physiologist Led HF clinic was implemented.

A BSE accredited physiologist with health assessment training/qualification, carried out an echocardiogram, ECG and clinical assessment within a single appointment. Preliminary outcome decisions made by the physiologist were then reviewed by the supervising HF consultant of the week.

**Purpose:** Evaluate the effectiveness and outcomes of the clinic aiming to ensure timely clinical assessment at the point of echocardiography and reduce the timeframe between echocardiography results and clinical decision making.

**Methods:** In a retrospective service evaluation, the clinic outcome and time from scan to outcome correspondence was compared between the clinic pathway (n = 38) and current pathway (n = 23). (Fig. 1).

**Results:** Both groups were found to have similar outcomes (discharged back to GP / referred into follow-up clinics). (Fig. 2).

There was an almost five-fold reduction (24.74 days vs 5.5 days) in time from echocardiogram to decision and outcome letter for patient. (p < 0.001) (Fig. 3).

**Conclusion:** With the current demand on Echocardiography and HF departments to provide a timely diagnosis of HF this clinic pathway provides improved scan to outcome time, ensuring timely treatment for those that need it. There is potential for increasing the number of clinics and further expansion of the physiologist role.

**Fig. 1 (abstract ABS013):**
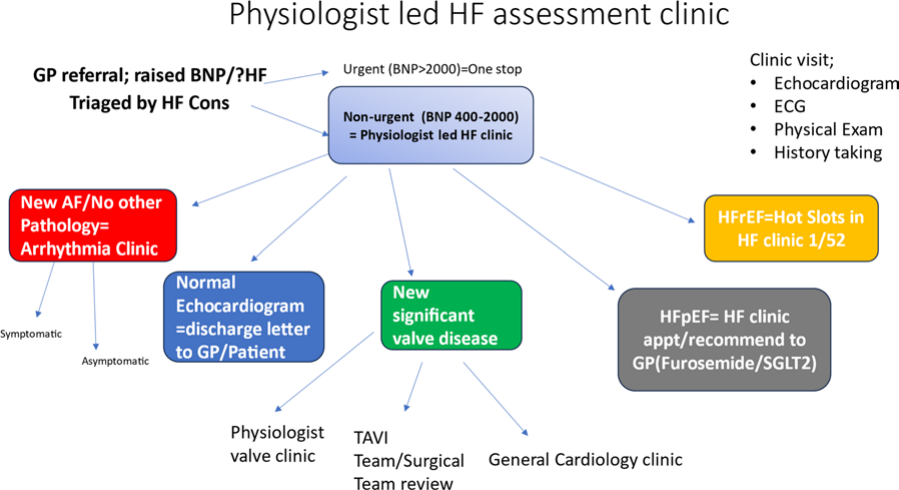
Illustration of clinic pathway

**Fig. 2 (abstract ABS013):**
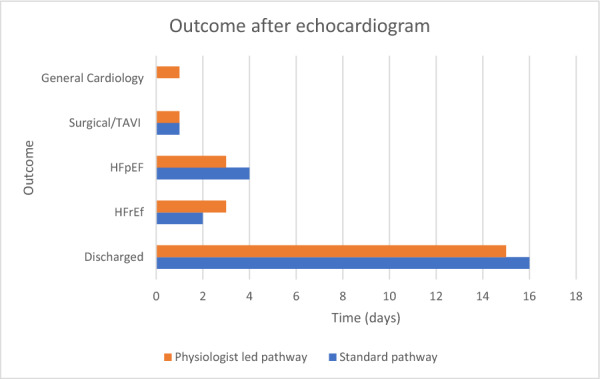
Outcomes after clinic appointment

**Fig. 3 (abstract ABS013):**
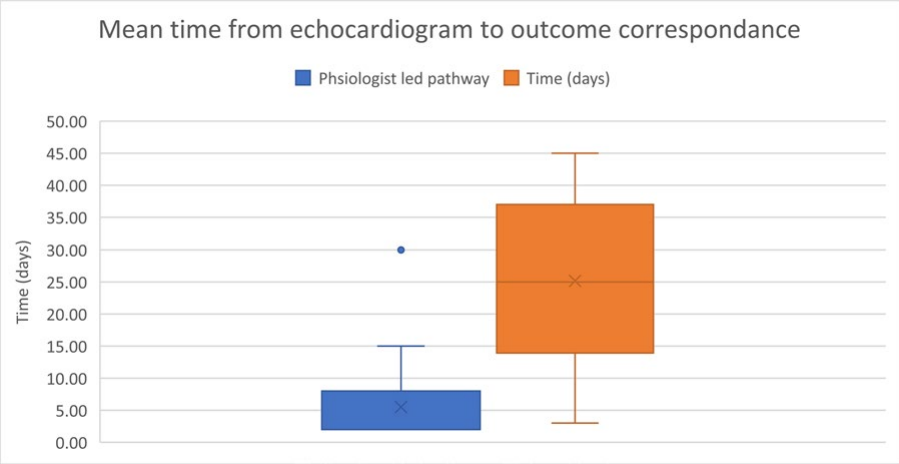
Mean time from scan to outcome correspondence

## ABS015: Straining to detect a large atrial shunt: Can LASr help?

### James Willis^1^, Thom Phillips^2^, Anil Gurung^1^, Joseph Sparey^1^, Daniel X. Augustine^1,3^

#### ^1^Royal United Hospitals NHS Foundation Trust, Bath. ^2^Bristol Bears Rugby Football Club, Bristol. ^3^Department for Health, University of Bath

*Echo Research & Practice* 2025, **12(Suppl 1):**ABS015

**Introduction:** High risk anatomy for patent foramen ovale (PFO) is well documented (for example a mobile inter atrial septum). Left atrial reservoir strain (LASr) is important in the assessment of left atrial mechanics. In a cohort of patients presenting with cryptogenic stroke for bubble contrast assessment, we used LA strain software to assess LASr at rest and at peak valsalva.

**Aim:** Can LASr help to identify those with a large shunt on provocation not appreciated on the resting echocardiogram?

**Method:** LASr was measured from the apical four chamber view on 58 patients (see Fig. 1). First, at the frame immediately prior to bubbles entering the RA at rest and then, the closest frame post RA opacification (at peak valsalva) in the NRS and LSV groups (Fig. 2).

Based on the test outcome, patients were split into one of three groups—No right to left shunt (NRS), large shunt with valsalva (LSV) and large resting shunt (no valsalva) (LRS). Paired analysis was performed.

**Results:** Comparing rest values between all three groups, demonstrated increased LASr values in the LRS group compared to both the NRS (p = 0.003) and LSV (p = 0.03) (see Table 1.0).

There was no difference in the LASr paired rest and valsalva values in the NRS group, however the LSV group demonstrated increased values post valsalva (24% vs. 27% p = 0.008).

**Discussion:** An increase in LASr at rest and with valsalva may be additive to identify PFOs with large shunts on provocation and thus higher risk of cryptogenic stroke. The addition of atrial mechanics assessment may be useful to anatomical analysis to identify high risk PFO features. Further prospective studies would be helpful to assess this theory.

**Fig. 1 (abstract ABS015):**
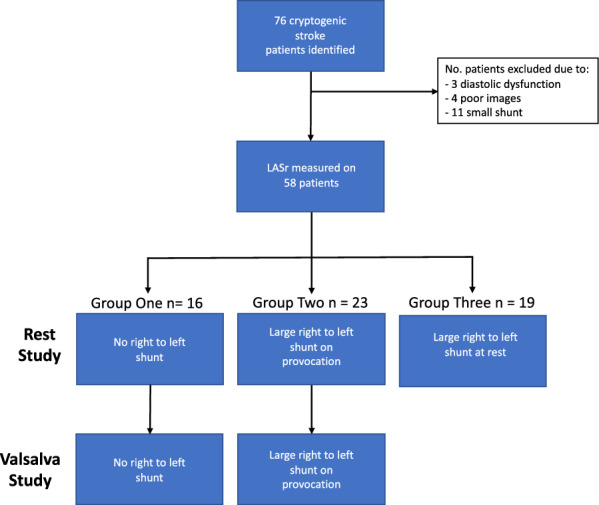
Patient Selection and testing criteria

**Fig. 2 (abstract ABS015):**
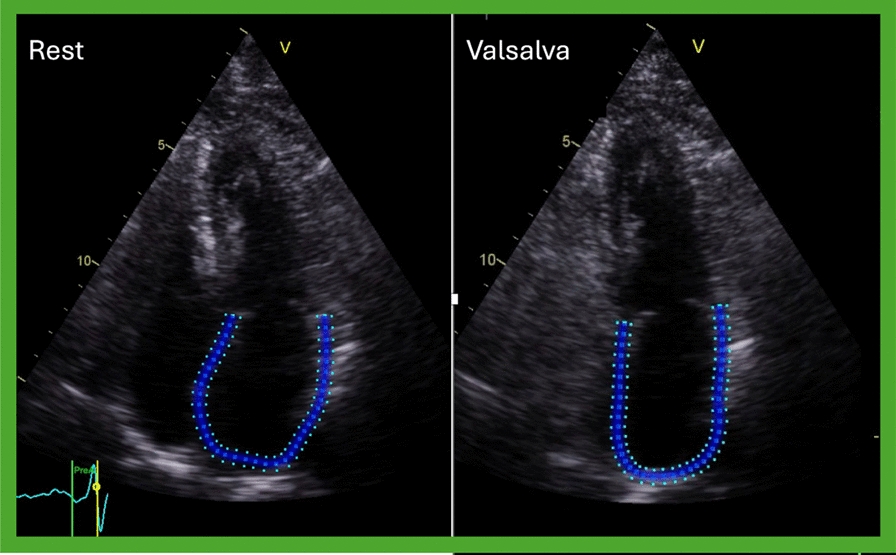
LASr acquired using dedicated strain software. This was undertaken at rest in all three groups and repeated during Valsalva in the LSV and NRS groups

**Table 1 (abstract ABS015):**
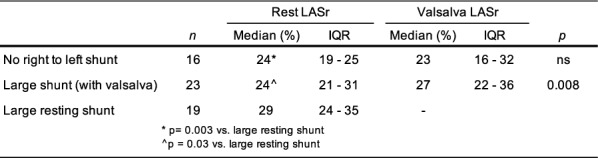
Paired and between groups median Left Atrial Reservoir Strain results

## ABS016: Impact of intensive blood pressure control on systolic function in children with CKD in the HOT-KID RCT

### Haotian Gu^1^, John Simpson^2^, Phil Chowienczyk^1^, Manish Sinha^1,2^

#### ^1^King’s College London; ^2^Evelina London Children’s Hospital

*Echo Research & Practice* 2025, **12(Suppl 1):**ABS016

**Introduction:** Relationship between blood pressure (BP) control and left ventricular (LV) systolic function in children with chronic kidney disease (CKD) is uncertain. The aim is to investigate whether achieving lower BP control yields a favourable impact on systolic function by performing an exploratory analysis utilising data from the HOT-KID randomised controlled trial (RCT) in children with CKD (ISRCTN25006406). ^1^

**Methods:** 124 children were randomised to standard (50th-75th percentile) or intensive (< 40th percentile) systolic blood pressure using office BP targets. Echocardiograms were performed at baseline and at follow-up visits. Systolic function was assessed from echocardiographic measures of ejection fraction (EF), Tissue Doppler lateral and septal S waves, Global Longitudinal Strain (GSL) and first-phase ejection fraction (EF1) by a blinded observer.

**Results:** At baseline, there was no significant difference of EF, S waves, GLS and EF1 between standard and intensive treatment groups. There was significant average annual rate of change in GLS (difference in means 0.39% per year, 95% CI: 0.107 to 0.68, P = 0.007) (Table 1a and Fig. 1a) and EF1 (difference in means − 0.97% per year, 95% CI − 1.44 to − 0.50, P < 0.001) (Table 1b and Fig. 1b) in the standard compared to the intensive treatment arm. However, the average annual changes in all other systolic function measures were similar between standard and intensive treatment groups.

**Conclusions:** Achieving lower blood pressure control has a favourable impact on early LV systolic function as measured by EF1 and longitudinal function as measured by GLS in children with CKD.

**Disclosures:** None.

**Table 1 (abstract ABS016):** Systolic function measures over time

A: Change in mean GLS (%) from baseline				
GLS (%)	Intensive arm	Standard arm	Difference (CI)	P
Change in per year, mean (95%, CI)	0.20 (0.003, 0.40)	0.60 (0.39, 0.80)	0.39 (0.107, 0.68)	**0.007**
Baseline (n = 64, 60)	− 17.2 (− 18.0, − 16.5)	− 17.1 (− 17.7, − 16.4)	0.15 (− 0.82, 1.12)	
Y1 (n = 51, 54)	− 16.4 (− 17.1, − 15.6)	− 16.5 (− 17.2, − 15.7)	− 0.12 (− 1.14, 0.90)	0.814
Y2 (n = 47, 47)	− 16.2 (− 16.9, − 15.5)	− 15.4 (− 15.9, − 14.8)	0.83 (− 0.07, 1.72)	0.069
Y3 (n = 36, 32)	− 16.9 (− 17.7, − 16.1)	− 16.0 (− 17.0, − 15.1)	0.86 (− 0.35, 2.08)	0.161
Y4 (n = 23, 22)	− 16.8 (− 17.8, − 15.8)	− 14.5 (− 15.7, − 13.3)	2.32 (0.80, 3.83)	**0.004**
Y5 (n = 11, 11)	− 15.7 (− 16.9, − 14.6)	− 14.7 (− 16.3, − 13.1)	1.00 (− 0.86, 2.88)	0.273

B: Change in mean EF1 (%) from baseline.				
EF1 (%)	Intensive arm	Standard arm	Difference (CI)	P
Change in per year, mean (95%, CI)	1.05 (0.73, 1.38)	0.08 (− 0.26, 0.42)	− 0.97 (− 1.44, − 0.50)	**<** **0.001**
Baseline (n = 64, 60)	19.8 (18.6, 21.0)	20.1 (18.8, 21.4)	0.30(− 1.45, 2.06)	
Y1 (n = 51, 54)	21.7 (20.3, 23.1)	20.6 (19.6, 22.1)	− 0.87 (− 2.73, 0.98)	0.353
Y2 (n = 47, 47)	22.3 (21.1, 23.5)	21.1 (19.7, 22.5)	− 1.17 (− 3.00, 0.66)	0.207
Y3 (n = 36, 32)	23.9 (22.4, 25.4)	20.8 (18.8, 22.8)	− 3.14 (− 5.58, − 0.72)	**0.012**
Y4 (n = 23, 22)	23.6 (22.0, 25.2)	19.3 (17.7, 21.0)	− 4.25 (− 6.47, − 2.03)	**<** **0.001**
Y5 (n = 11, 11)	22.8 (21.1, 24.6)	18.3 (16.1, 20.5)	− 4.55 (− 7.18, − 1.92)	**0.002**

P value denotes difference in means for change in each variable per year between two trial arms, standard vs. intensive for all the data. Means were estimated by use of a linear mixed effects model for repeated measures. At the final follow up visit the majority of patients had not reached 5 years of study participation, which accounts for the sharp decrease in numbers available for follow-up between year 4 and 5

**Fig. 1 (abstract ABS016):**
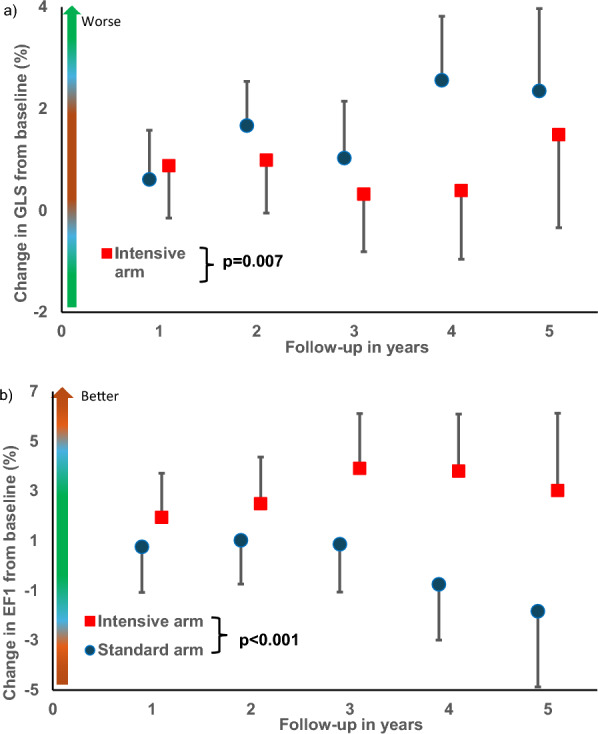
Myocardial fibrosis is associated with left ventricular early systolic function in a pressure overload inducted heart failure rat model

## ABS017: Myocardial fibrosis is associated with left ventricular early systolic function in a pressure overload inducted heart failure rat model

### Shukun He^1^, Jing Wang^1^, Mingxing Xie^1^, Haotian Gu^2^

#### ^1^Union Hospital, Tongji Medical College, Huazhong University of Science and Technology, China; ^2^King’s College London, United Kingdom

*Echo Research & Practice* 2025, **12(Suppl 1):**ABS017

**Introduction:** The induction of pressure overload instigates myocardial fibrosis (MF) and diminishes myocardial compliance, precipitating left ventricular dysfunction, and eventual heart failure (HF). Timely evaluation of myocardial remodelling and fibrosis in HF holds significant clinical implications for patient management. First-phase ejection fraction (EF1) has demonstrated sensitivity in detecting left ventricular early systolic dysfunction. This study aims to explore the relationship between EF1 and MF in a transverse aortic constriction rat model.

**Methods:** A pressure overload heart failure rat model was established through minimally invasive transverse aortic constriction. Echocardiography was conducted weekly, with an equivalent number of rats randomly selected from each group to measure EF1, myocardial strain, and conventional parameters. Subsequently, rat hearts underwent haematoxylin and eosin staining and Masson's trichrome staining analysis.

**Results:** Compared to the control and sham-operated groups, the HF group exhibited a significant reduction in EF1 starting from the second week post-operation, with further declines observed over time (P < 0.05) (Fig. 1). Left ventricular global longitudinal strain (GLS) and ejection fraction (EF) were impaired by the third week post-operation (P < 0.05) (Fig. 1), while MF and hypertrophy manifested as early as the second week post-operation (P < 0.05). Linear regression analysis demonstrated a robust association between EF1 and MF, after adjusting for GLS, EF, global circumferential strain (GCS), and global radial strain (GRS) (P < 0.001). Diagnostic efficiency assessments revealed that the area under the receiver operating characteristic curve of EF1 for detecting MF was the highest (AUC = 0.89), surpassing that of GLS and EF. (Fig. 2).

**Conclusions:** EF1 is a sensitive non-invasive marker of early left ventricular systolic dysfunction and a promising indicator for identifying MF at early stage of heart failure. Incorporating EF1 into routine clinical assessments may facilitate timely interventions to mitigate myocardial fibrosis progression.

**Disclosures:** None.

**Fig. 1 (abstract ABS017):**
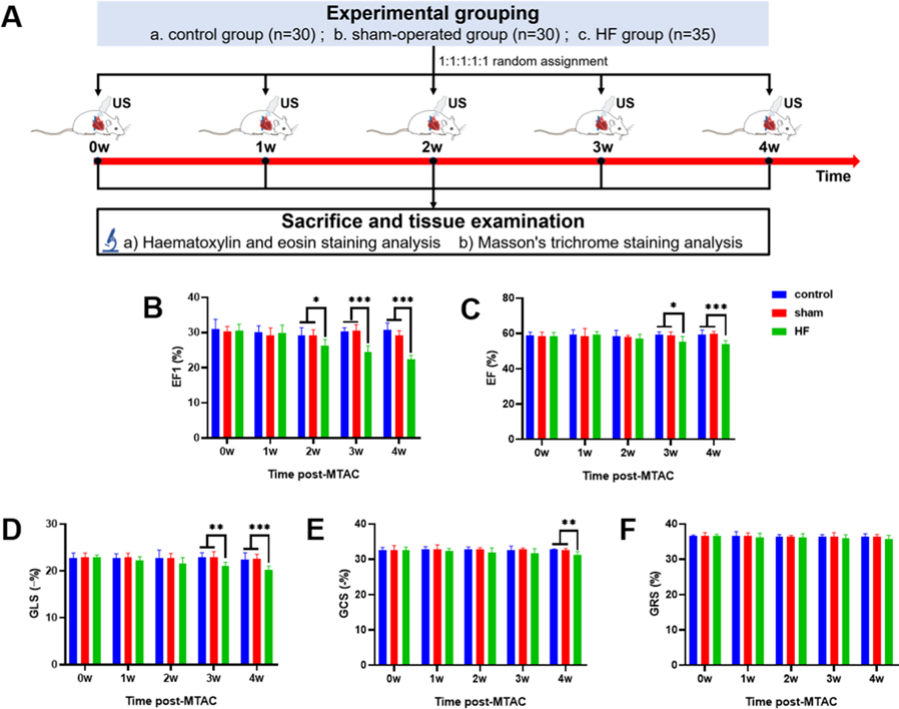
(A): Experimental grouping and time-line of echocardiography and pathological examination of rats; (B): EF1, first-phase ejection fraction; (C): EF, left ventricular ejection fraction; (D): GLS, global longitudinal strain; (E): GCS, global circumferential strain; (F): GRS, global radial strain. Graphs represent mean ± SD. *, P < 0.05; **, P < 0.01; ***, P < 0.001

**Fig. 2 (abstract ABS017):**
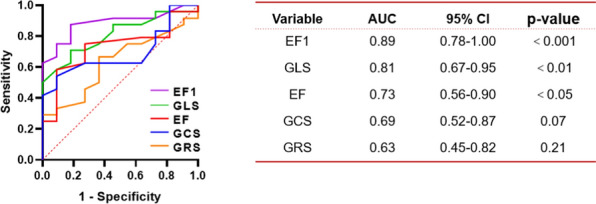
ROC Curve Analysis. Receiver operating characteristic curve of echocardiographic parameters for detecting the moderate and severe myocardial fibrosis in HF rats. AUC, area under the curve; EF1, first-phase ejection fraction; GLS, global longitudinal strain; GCS, global circumferential strain; GRS, global radial strain; EF, left ventricular ejection fraction

## ABS018: Exercise capacity among adults aged 19–80 referred for supine bike stress echocardiography—time or effort?

### Joseph Christopher Flynn^1^, Bethany Elliott^1^, Keith Pearce^2^, Martin Stout^2^, Anita Macnab^2^, Laura E. Dobson^2^

#### ^1^University of Manchester Medical School, Manchester, UK; ^2^Manchester University NHS Foundation Trust, Manchester, UK

*Echo Research & Practice* 2025, **12(Suppl 1):**ABS018

**Background/introduction:** Current normal reference ranges for exercise capacity for bicycle stress echocardiography (BSE) are unknown.

**Purpose:** The aim of this study is to investigate if BSE is an adequate measurement of peak exercise capacity throughout all age cohorts, and to determine if normal reference values for rate pressure product (RPP) or exercise time are a more suitable guide to use for peak exercise performance.

**Methods:** 776 patients who underwent the WHO 25W bike ergometer protocol were screened. 516 were excluded due to pre-specified exclusion criteria. Anonymised data from 260 patients was analysed, recording data regarding both baseline and peak measurements of heart rate, systolic and diastolic blood pressure, workload in Watts, RPP and total exercise time.

**Results:** The peak heart rate in women was 10.2% lower on average compared to men across all age cohorts (P < 0.05). The mean peak systolic blood pressure for men and women combined increased with age (< 40—152.9 mmHg (± 27.3), 40–49—173.2 mmHg (± 29.3), 50–59—181.6 mmHg (± 30.6), 60–69—183.8 mmHg (± 28.7), > 70- 188.6 mmHg (± 23.9)). With progressive age, the mean total exercise time decreased (< 40—609.4 s (± 157.9), 40–49—584.6 s (± 183.5), 50–59—554.1 s (± 140.4), 60–69- 497.6 s (± 170.1), > 70—475.3 s (± 126.1)). Total exercise time in women was 20.5% lower on average than that of men (P < 0.0005). There was a statistically significant difference between men and women for all parameters defined (P < 0.05). The reduced exercise time in older patients was offset by the greater peak blood pressure in this group, meaning the RPP was similar across all age groups for men and women (P = 0.14 for men, P = 0.27 for women).

**Conclusions:** This study established normal reference values for peak RPP and total exercise time, and demonstrated that as patients age, although exercise time reduces, the RPP remains the same. Therefore, RPP is the most suitable representation of an individual’s peak exercise performance, whereas exercise time is an age dependant representation of peak performance.

**Table 1 (abstract ABS018):** Physiologic Responses at Maximal Exertion for Men by Age. Data presented as mean (± SD). HR: heart rate, SBP: systolic blood pressure, DBP: diastolic blood pressure, RPP: rate pressure product

Age range	< 40	40–49	50–59	60–69	> 70
Number of Subjects	16	22	48	40	27
Peak HR (bpm)	155.9 (10.1)	149.6 (7.0)	144.1 (5.2)	136 (7.5)	127.6 (5.4)
SBP (mmHg)	167.3 (26.8)	183.5 (27.1)	184.2 (30.1)	187.1 (28.7)	193.6 (26.1)
DBP (mmHg)	91.8 (17.4)	90.7 (12.5)	94.4 (12.5)	93.5 (14.7)	91.3 (16.2)
RPP	26042.3 (4309.9)	27465.5 (4310.7)	26543.1 (4387.8)	25442.7 (4176.6)	24674.3 (3265.1)
Exercise Time (s)	651.0 (177.8)	629.0 (179.0)	589.0 (142.9)	556.0 (164.4)	498.0 (129.5)
Workload (Watts)	135.9 (45.7)	149.8 (38.1)	126.8 (29.2)	106.9 (27.7)	93.7 (28.2)
METs	9.9 (1.3)	9.8 (1.3)	9.5 (0.6)	9.3 (0.7)	9.0 (0.7)

**Table 2 (abstract ABS018):** Physiologic Responses at Maximal Exertion for Women by Age. Data presented as mean (± SD). HR: heart rate, SBP: systolic blood pressure, DBP: diastolic blood pressure, RPP: rate pressure product

Age range	< 40	40–49	50–59	60–69	> 70
Number of Subjects	16	19	19	37	16
Peak HR (bpm)	156.9 (7.3)	152.3 (9.7)	144.9 (5.0)	140.2 (7.8)	131.9 (5.8)
SBP (mmHg)	138.6 (19.5)	161.3 (27.7)	174.9 (31.7)	180.2 (28.7)	180.0 (16.2)
DBP (mmHg)	83.1 (15.3)	85.8 (14.7)	90.7 (15.4)	90.0 (20.9)	90.6 (11.5)
RPP	21721.4 (3141.9)	24662.1 (5011.8)	25336.2 (4582.7)	25096.8 (4373.6)	23785 (2626.8)
Exercise Time (s)	567.0 (126.8)	532.0 (179.3)	465.0 (85.7)	433.0 (154.1)	436.0 (113.6)
Workload (Watts)	99.7 (26.9)	86.3 (23.5)	81.7 (19.8)	74.3(22.5)	68.8 (19.0)
METs	9.3 (0.9)	9.2 (0.8)	8.9 (0.6)	8.7 (0.7)	8.9 (0.7)

**Fig. 1 (abstract ABS018):**
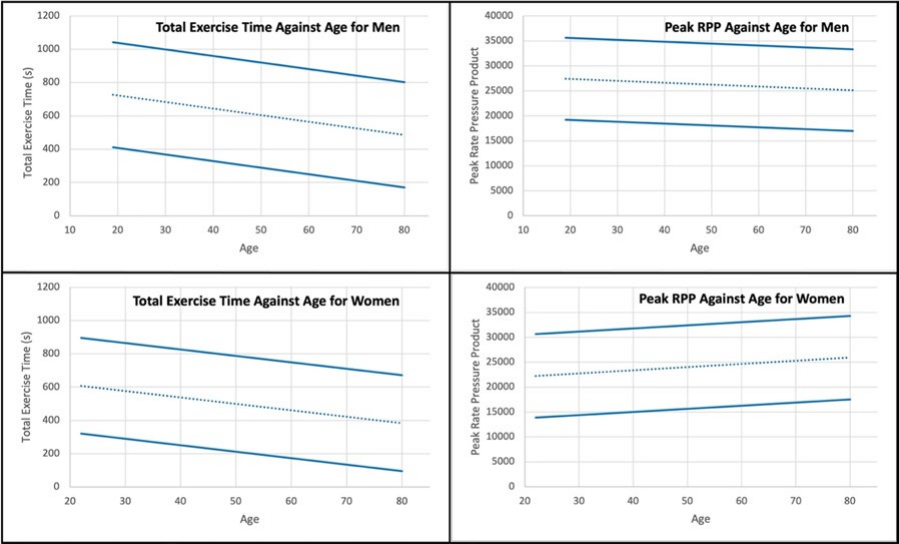
Graphs demonstrating total exercise time in minutes and peak rate pressure product for men and women according to age

## ABS019: What is the fate of patients with cavity dilatation on bike stress echocardiography?

### Bethany Elliott^1^, Joseph Christopher Flynn^1^, Keith Pearce^2^, Martin Stout^2^, Anita Macnab^2^, Laura E. Dobson^2^

#### ^1^University of Manchester Medical School, Manchester, UK; ^2^Manchester University NHS Foundation Trust, Manchester, UK

*Echo Research & Practice* 2025, **12(Suppl 1):**ABS019

**Background/introduction:** Cavity dilatation (CD) is occasionally observed during exercise stress echocardiography (ESE). Mechanisms are poorly understood (Fig. 1); these patients have not previously been studied in the published literature.

**Purpose:** This study aims to characterise patients with left ventricle (LV) CD and a fall in left ventricular ejection fraction during bike ESE.

**Methods:** 653 patients who underwent bike ESE were evaluated. Those with exercise-induced CD (defined as increased cavity size and a fall in left ventricular ejection fraction) were evaluated (n = 29). A control group (n = 37) of patients with hypertensive response and a normal ESE were also evaluated.

**Results:** 33/653 (5.1%) patients had exercise-induced CD of the LV, of which full data was available for 29 patients. 15/29 (51.7%) of those with CD had significant underlying coronary artery disease (CAD). There was a hypertensive response to exercise in 5/15 (33%) CAD-CD patients and 12/14 (86%) with non-obstructed coronary arteries (NCA-CD). Baseline and ESE characteristics of the two groups can be seen in Table 1. NCA-CD patients had higher diastolic blood pressure (NCA-CD 109 ± 17mmHg vs CAD-CD 96 ± 16mmHg, p = 0.039), rate-pressure product (NCA-CD 28,623 ± 4474 vs CAD-CD 23,649 ± 4763, p = 0.007) and dyspnoea frequency at peak exercise (NCA-CD 35.7% vs CAD-CD 6.7%, p = 0.054). CAD-CD patients experienced more severe chest pain than NCA-CD (CAD-CD 53.3% vs. NCA-CD 14.3%, p = 0.027). No significant differences were observed between patients with and without CD in the setting of a hypertensive response (Table 2).

**Conclusions:** CD is relatively common during bike ESE and is due to significant CAD in around half of patients. NCA-CD is associated with higher rate-pressure product, diastolic blood pressure and dyspnoea at peak exercise. Severe chest pain at peak exercise strongly indicates CAD-CD.

**Fig. 1 (abstract ABS019):**
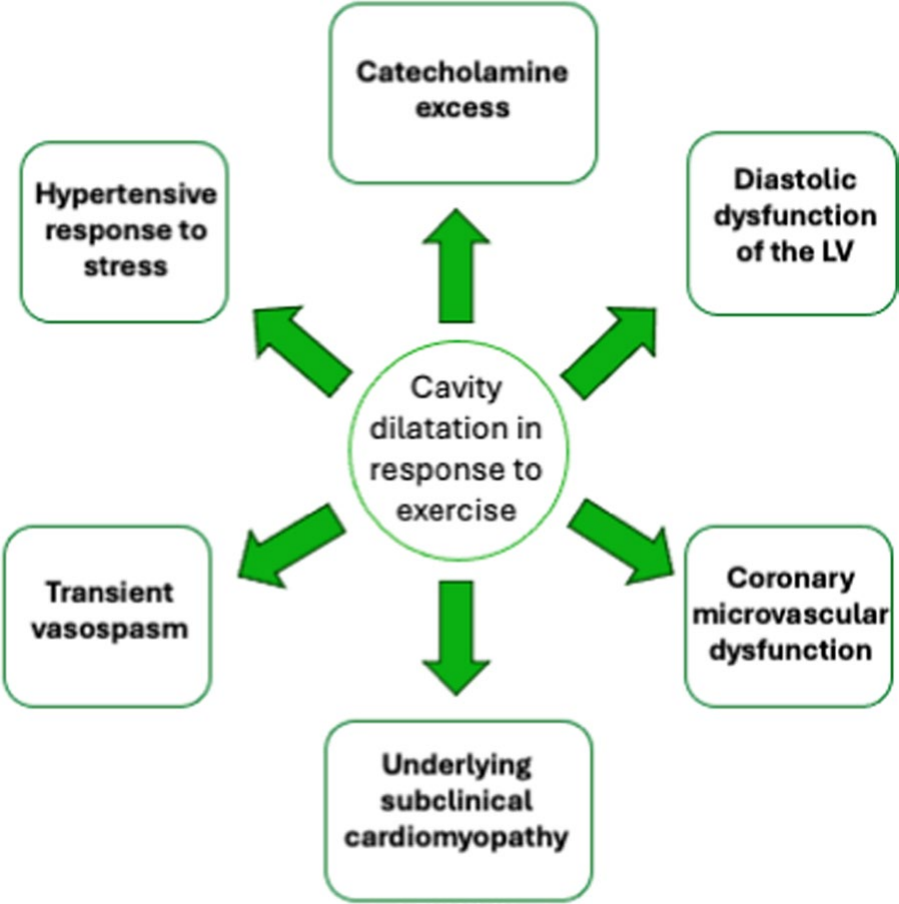
Proposed mechanisms behind an abnormal LV response (cavity dilatation and/or a reduction in left ventricular ejection fraction) during exercise. LV = Left ventricle

**Table 1 (abstract ABS019):** Patient characteristics and bike stress echocardiography parameters in patients with cavity dilatation, comparing patients with underlying coronary artery disease (CADCD) to those with non-obstructed coronary arteries (NCA-CD). Data is expressed as mean ± standard deviation or as a proportion of the population (%), with n = the number of patients. * indicates statistically significant results (p < 0.05)

Variable	CAD-CD (n = 15)	NCA-CD (n = 14)	p-value
**Patient characteristics**			
**Age**	63 ± 8.2	59.5 ± 7.8	p = 0.257
**Females**	33.3% (n = 5)	64.3% (n = 9)	p = 0.096
**BMI**	28.5 ± 5.1	27.2 ± 4.7	p = 0.457
**No. on HTN therapy**	60% (n = 9)	57.2% (n = 8)	p = 0.876
**Type 2 diabetes**	26.7% (n = 4)	14.3% (n = 2)	p = 0.411
**Dyslipidaemia**	60% (n = 9)	35.7% (n = 5)	p = 0.191
**Smoker or ex-smoker**	60% (n = 9)	64.3% (n = 9)	p = 0.812
**FH of CAD**	60% (n = 9)	64.3% (n = 9)	p = 0.812
**PMH of anxiety or depression**	33.3% (n = 5)	42.9% (n = 6)	p = 0.597
**Echocardiography parameters**			
**Resting LV systolic dysfunction **	20% (n = 3)	14.3% (n = 2)	p = 0.684
**Resting HR**	83 ± 20	77 ± 13	p = 0.354
**Peak HR **	128 ± 16	138 ± 14	p = 0.074
**Change in HR**	44 ± 61	61 ± 14	p = 0.009*
**Resting SBP**	161 ± 27	149 ± 31	p = 0.277
**Peak SBP**	186 ± 34	208 ± 27	p = 0.069
**Change in SBP**	26 ± 32	59 ± 33	p = 0.009*
**Resting DBP**	87 ± 11	78 ± 14	p = 0.079
**Peak DBP**	96 ± 16	109 ± 17	p = 0.039*
**Change in DBP**	9 ± 13	31 ± 20	p = 0.002*
**Resting RPP**	13,086 ± 2431	11,479 ± 2925	p = 0.118
**Peak RPP**	23,649 ± 4763	28,623 ± 4474	p = 0.007*
**Exercise time (mins:secs)**	06:43 ± 03:12	07:48 ± 02:59	p = 0.374
**Symptoms during test**			
**Presence of any symptoms**	66.7% (n = 10)	64.3% (n = 9)	p = 0.893
**Chest pain **	60% (n = 9)	28.6% (n = 4)	p = 0.089
**Dyspnoea**	6.7% (n = 1)	35.7% (n = 5)	p = 0.054*
**Severe chest pain**	53.3% (n = 8)	14.3% (n = 2)	p = 0.027*

**Table 2 (abstract ABS019):** Patient characteristics and bike stress echocardiography parameters in patients with a hypertensive response to exercise, comparing patients with non-obstructed coronary arteries and cavity dilatation (NCA-CD) to those with a normal left ventricular response to exercise (negative ESE). Data is expressed as mean ± standard deviation or as a proportion of the population (%), with n = the number of patients

**Variable**	**NCA-CD & hypertensive response (n=12)**	**Negative ESE & hypertensive response (n=37)**	**p-value**
**Patient characteristics**			
**Age**	60.5 ± 8	60.8 ± 10.8	p=0.927
**Females**	66.7% (n=8)	43.2% (n=16)	p=0.158
**BMI**	27.8 ± 4.8	30.2 ± 5.1	p=0.159
**No. on HTN therapy **	58.3% (n=7)	62.2% (n=23)	p=0.813
**Type 2 diabetes**	16.7% (n=2)	16.2% (n=6)	p=0.971
**Dyslipidaemia**	33.3% (n=4)	37.8% (n=14)	p=0.779
**Smoker or ex-smoker**	58.3% (n=7)	40.5% (n=15)	p=0.282
**FH of CAD**	58.3% (n=7)	54.1% (n=20)	p=0.796
**PMH of anxiety or depression**	50% (n=6)	24.3% (n=9)	p=0.094
**Echocardiography parameters**			
**Resting HR**	76 ± 14	82 ± 15	p=0.247
**Peak HR **	137 ± 15	135 ± 13	p=0.671
**Change in HR**	61 ± 14	53 ± 14	p=0.091
**Resting SBP**	152 ± 32	152 ± 20	p=0.967
**Peak SBP**	213 ± 26	219 ± 20	p=0.378
**Change in SBP**	61 ± 34	67 ± 28	p=0.545
**Resting DBP**	79 ± 15	85 ± 13	p=0.221
**Peak DBP**	112 ± 15	110 ± 19	p=0.728
**Change in DBP**	33 ± 20	25 ± 20	p=0.249
**Resting RPP**	11569 ± 3066	12694 ± 3186	p=0.289
**Peak RPP**	29125 ± 4466	28581 ± 5175	p=0.746
**Exercise time (mins:secs)**	08:04 ± 03:02	07:43 ± 03:14	p=0.749
**Symptoms during test**	66.7% (n=8)	43.2% (n=16)	p=0.158
**Chest pain during test**	25% (n=3)	13.5% (5)	p=0.350
**Dyspnoea during test**	41.7% (n=5)	27% (10)	p=0.339

## ABS021: Clinical audit: discordant and concordant transthoracic echocardiographic criteria in the diagnosis of severe aortic stenosis

### Bishoy Bishay, Thomas Martin

#### Cardiology Department at Ninewells Hospital and Medical School, NHS Tayside, Dundee, UK

*Echo Research & Practice* 2025, **12(Suppl 1):**ABS021

**Background:** Diagnosing severe aortic stenosis (AS) via transthoracic echocardiography requires careful consideration to ensure appropriate clinical response, especially when aortic valve velocity (AV Vmax) is below 4 m/s (Discordant criteria).

**Purpose:** Audit departmental labelling criteria for severe AS.

**Methods:** Two-cycle audit was conducted over 10 months. First cycle involved 100 patients labelled severe AS, followed by a presentation of the results, then 72 patients in second cycle. We examined LV function, AV area (AVA), AV Vmax, AV mean gradient, symptoms, AV calcium score, cardiology review, referral for intervention, and outcomes.

**Results:** After first cycle results, (1) We highlighted with the team common pitfalls in AVA measurements as per BSE guidelines. (2) Agreed to re-phrase discordant group reports to “Visual appearance and calculated AVA suggest severe AS, although Vmax and gradients are indicative of moderate AS”. Second cycle: Concordant group increased from 59 to 68%. (Fig. 1). Discordant group with normal/mild LVSD decreased from 19% to 6.9%. Mean Vmax in that group increased from 3.5 to 3.7 m/sec. 60% of discordant group had the agreed re-phrase added to their reports.

**Fig. 1 (abstract ABS021):**
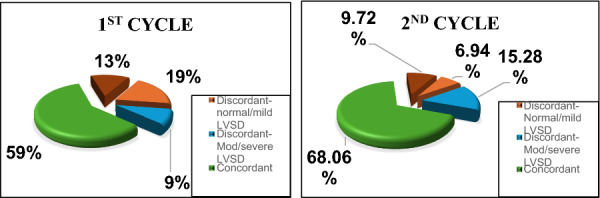
Pie charts showing results of 1st and 2nd cycles

**Conclusions:** First cycle, 28% were labelled by AVA alone and 82% of them were not indicated for valve intervention. Patients may be denied important non-cardiac surgeries based on the label of severe AS while awaiting cardiology review. Interventions after 1st cycle led to precise AVA calculations and reduced percentage of discordant group with preserved LV function from 19 to 6.9%.

## ABS023: Prognostic value of first-phase ejection fraction: a systematic review

### Rahaf Alshehri, Samuel Burden, Tanisha Rajah, Phil Chowienczyk, Haotian Gu

#### King’s College London

*Echo Research & Practice* 2025, **12(Suppl 1):**ABS023

**Introduction:** First phase ejection fraction (EF1) is a simple but robust measure of early left ventricular systolic function. This study is to assemble evidence that EF1 is an accurate and sensitive marker in predicting cardiovascular outcomes, compared to ejection fraction (EF) and global longitudinal strain (GLS).

**Methods:** A systematic review of the literature was conducted according to the Preferred Reporting Items Systematic reviews and Meta-Analyses (PRISMA) protocol. An electronic search of all English publications from PubMed and Embase via Ovid databases was undertaken. All studies which compared EF1 against EF and GLS in predicting cardiovascular outcomes were included. The primary outcome was all-cause mortality, and the secondary outcome was a composite of death, hospitalization due to heart failure, cardiac intervention or coronary ischemic event. Inclusion criteria: All EF1 calculations in regards of cardiovascular system, whereas EF1 used as a predictor of major adverse cardiac events and mortality.

**Results:** Review was conducted from 10 published full research articles that included 2538 adults comprising 2 prospective and 8 retrospective observational studies between 2017 and 2024 (Fig. 1). The underlying cardiac diseases were aortic stenosis, heart failure, heart transplant and COVID-19. Overall, EF1 was found to be an independent predictor of adverse events.

**Conclusions:** Our systematic review suggested that the prognostic value of EF1 appears to be superior to EF and GLS for predicting adverse cardiovascular events. A lower EF1 (< 25%) was associated with poorer outcomes.

**Disclosures:** HG and PC are named on a patent of EF1.

**Fig. 1 (abstract ABS023):**
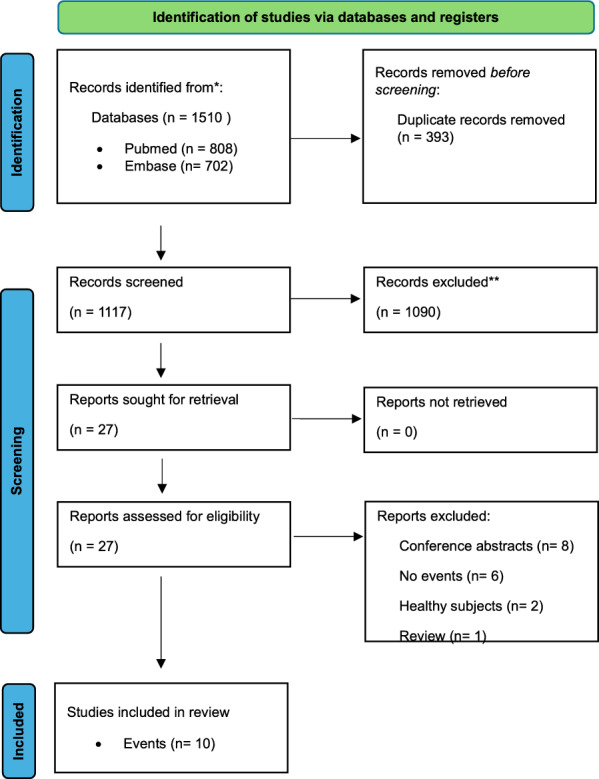
Flow chart identification of studies via databases and registers

## ABS024: Acute alterations in left ventricular structure and mechanics following Interventricular CRT optimisation

### Rhiannon Peters^1^, Mathuri Partheban^2^, Nurul H. Abdul Samad^2^, Maria F. Paton^1,2^

#### ^1^Leeds Teaching Hospitals NHS Trust, Leeds, UK; ^2^Leeds Institute of Cardiovascular and Metabolic Medicine, University of Leeds, Leeds, UK

*Echo Research & Practice* 2025, **12(Suppl 1):**ABS024

**Background:** Cardiac resynchronisation therapy (CRT) is a well-established treatment for patients with left ventricular (LV) systolic dysfunction and ventricular conduction delay. Individualised optimisation has been shown to improve CRT response rates. Non-invasive myocardial work (MW) quantification is an emerging echocardiographic measure which overcomes some limitations of traditional imaging markers of cardiac function as it is somewhat adjusted for loading factors, hence may be a useful in driving CRT optimisation.

**Aim:** To assess LV size, function, and MW parameters at varying V-V offset intervals during CRT device optimisation.

**Methods:** A service evaluation was conducted with data from 10 patients attending the combined heart failure and device (CHAD) clinic at a single tertiary centre. CRT optimisation included altering V-V offsets as standard care. Echocardiographic images were obtained for each V-V configuration 1 min after reprogramming and were used to calculate LV end diastolic and systolic volumes, ejection fraction (EF), and MW. The primary outcome was within-patient differences in cardiac size and function across V-V intervals.

**Results:** No statistically significant within-patient differences were observed in LV size or function acutely following V-V reprogramming. A non-significant increase in Global Work Index was observed for left–right interventricular programming (*p* = 0.84). Intra-observer agreement was excellent (ICC > 0.90, *p* < 0.05) for all MW parameters, except for Global Wasted Work (ICC = 0.36, *p* = 0.22) (Table 1).

**Conclusion:** Quantification of echocardiographic measurements during CRT optimisation is feasible and reproducible. There are limited measurable acute changes in LV size or global function to direct interventricular delay optimisation, although, MW index may have the potential to provide useful data and may indicate left to right configurations are beneficial in most patients.

**Table 1 (abstract ABS024):** Change in outcome variables during CRT optimisation: comparison of LV-RV, RV-LV, and simultaneous interventricular programming

Outcome	Settings	Mean [95% Confidence Interval]	P value
LVEF (%)	LV-RV	36.11 [29.51, 42.72]	0.54
	RV-LV	35.78 [29.25, 42.31]	
	Simultaneous	35.33 [29.18, 41.48]	
LVEDV (mL)	LV-RV	200.78 [142.79, 258.76]	0.93
	RV-LV	214.33 [150.33, 278.33]	
	Simultaneous	201.22 [149.42, 253,02]	
LVESV (mL)	LV-RV	130.11 [84.98, 175.24]	0.73
	RV-LV	141.67 [88.09, 195.24]	
	Simultaneous	131.67 [91.63, 171.71]	
LVCO (L/min)	LV-RV	4.69 [3.61, 5.77]	0.08
	RV-LV	4.72 [3.92, 5.52]	
	Simultaneous	4.63 [3.55, 5.71]	
LVSV (mL)	LV-RV	70.56 [50.90, 90.21]	0.74
	RV-LV	72.33 [57.70, 86.97]	
	Simultaneous	69.33 [50.87, 87.79]	
GWE (%)	LV-RV	76.78 [68.44, 85.12]	0.27
	RV-LV	74.56 [64.83, 84.28]	
	Simultaneous	79.78 [72.91, 86.65]	
GWI (mmHg%)	LV-RV	893.67 [378.63, 1408.70]	0.84
	RV-LV	816.33 [455.119, 1177.55]	
	Simultaneous	865.78 [547.29, 1184.27]	
GCW (mmHg%)	LV-RV	1151.00 [609.86, 1692.14]	0.83
	RV-LV	1207.67 [685.79, 1729.54]	
	Simultaneous	1178.00 [845.68, 1510.32]	
GWW (mmHg%)	LV-RV	251.11 [123.88, 378.35]	
	RV-LV	330.11 [172.60, 487.63]	0.91
	Simultaneous	258.89 [149.66, 368.12]	

Values are mean change [95% confidence intervals]; 95% significance shown in bold, *Denotes significance (*p* < 0.05). LV; left ventricle, RV; right ventricle, LVEF; left ventricular ejection fraction, LVEDV; left ventricular end-diastolic volume, LVESV; left ventricular end systolic volume, LVCO; left ventricular cardiac output, LVSV; left ventricular stroke volume, GWE; global work efficiency, GWI; global work index, GCW; global constructive work, GWW; global wasted work

**Fig. 1 (abstract ABS024):**
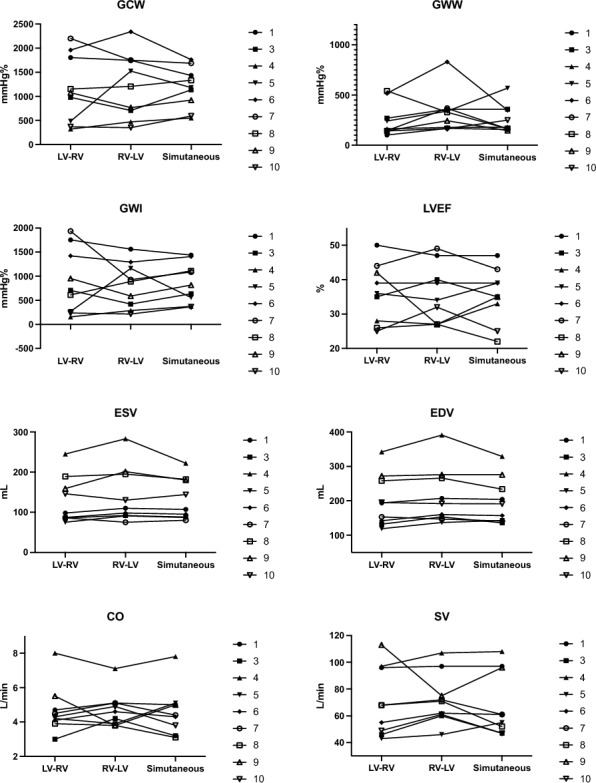
Individual values at each VV setting. LV; left ventricle, RV; right ventricle, LVEF; left ventricular ejection fraction, EDV; left ventricular end-diastolic volume, ESV; left ventricular end systolic volume, CO; left ventricular cardiac output, SV; left ventricular stroke volume, GWE; global work efficiency, GWI; global work index, GCW; global constructive work, GWW; global wasted work

**Fig. 2 (abstract ABS024):**
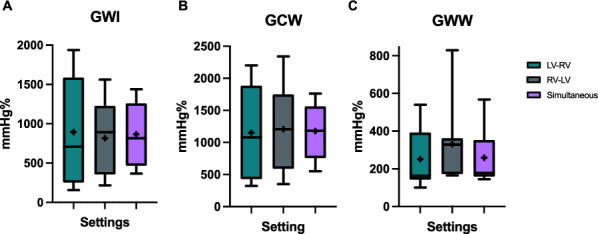
Mean (+) values at each VV setting for A) GWI, B—GCW and C—GWW. LV; left ventricle, RV; right ventricle, GWI; global work index, GCW; global constructive work, GWW; global wasted work

## ABS025: Left ventricular mechanics in conduction system pacing versus right ventricular pacing

### Mathuri Partheban^1^, Nurul H. Abdul Samad^1^, Shumaila Ahmad^1^, Maria F. Paton^1,2^

#### ^1^University of Leeds, Leeds Institute of Cardiovascular and Metabolic Medicine, Leeds, UK; ^2^Leeds Teaching hospitals Trust, Leeds, UK

*Echo Research & Practice* 2025, **12(Suppl 1):**ABS025

Funding Acknowledgement: NIHR Clinical Lectureship (NIHR301225).

**Introduction:** For 60 years, right ventricular pacing (RVP) has dominated treatment options for bradyarrhythmia but has been associated with adverse left ventricular (LV) remodelling and increased heart failure risk. Conduction system pacing (CSP) is a potential alternative pacing approach, though its effectiveness in reducing LV dyssynchrony is uncertain. Echocardiography is an essential tool for providing insights into LV function and mechanics.

**Aim:** To compare mechanical dyssynchrony in patients receiving CSP or RVP using modern echocardiographic myocardial work (MW) measurements.

**Methods:** Patients with atrioventricular block requiring pacemakers were prospectively recruited from October 2022 to October 2023 at a UK tertiary hospital. Patients receiving CSP were age and sex matched with patients receiving RVP. Data were collected at pre-implant (baseline), 0–6 weeks post-implant (acute), and 6 months post-implant (mid-term). The primary outcome was within-patients changes in septal and lateral MW indices between the groups.

**Results:** Sixteen patients (CSP n = 8, RVP n = 8), 76% male with a mean age 65 (± 11) years, were included for analysis. After a median follow-up of 27 weeks (IQR: 23–31), A significant time interaction effect in septal MW index was found between CSP and RVP groups (p = 0.04). Septal (1411 ± 198 to 1446 ± 178 mmHg%) and lateral work (1835 ± 263 to 1890 ± 243 mmHg%) was similar at baseline and across follow-up in those receiving CSP, whereas RVP patients had a significant decrease in septal work (1621 ± 198 to 954 ± 178 mmHg%) and an increase in lateral work (1283 ± 263 to 1788 ± 243 mmHg%) over time.

**Conclusions:** Regional LV wall analysis is required beyond global LV systolic function assessment to assess early changes in cardiac mechanics following pacemaker therapy initiation. Our data suggest CSP better preserves septal MW compared to RVP in age and sex matched individuals, potentially offering mechanistic insights into the effects of various bradycardia pacing strategies.

**Table 1 (abstract ABS025):** Baseline characteristics of CSP and RVP groups

	Total Cohort *n* *=* *16*	CSP Group *n* *=* *8*	RVP Group *n* *=* *8*	p-value
Age (years)	65 (± 11)	64 (± 11)	66 (± 12)	0.73
Sex (male)	12 (76)	6 (38)	6 (38)	0.72
Height (cm)	173 (± 9)	173 (± 11)	173 (± 8)	0.88
Weight (kg)	93 (± 25)	85 (± 16)	101 (± 30)	0.21
Blood pressure				
Systolic (mmHg)	145 (± 27)	142 (± 16)	147 (± 36)	0.76
Diastolic (mmHg)	76 (± 17)	76 (± 13)	76 (± 15)	1.00
Co-morbidities				
Atrial fibrillation	2 (11)	0 (0)	2 (11)	0.24
Type II Diabetes Mellitus	5 (31)	1 (6)	4 (25)	0.14
Cerebrovascular Accident	2 (12)	1 (6)	1 (6)	0.77
Diagnosed Ischaemic heart diseases	1 (6)	0 (0)	1 (6)	0.50
MI	0 (0)	0 (0)	0 (0)	–
PCI	1 (6)	0 (0)	1 (6)	0.50
CABG	0 (0)	0 (0)	0 (0)	–
Medical therapy				
β-blockers	6 (37)	1 (6)	5 (31)	0.06
ACE-inhibitors	7 (44)	4 (25)	3 (19)	0.50
Diuretics	5 (32)	3 (19)	2 (13)	0.50
Blood investigation				
BNP (pg/dl)	1252 (± 2139)	1420 (± 2715)	1060 (± 1412)	0.76
Pacing system				
Dual chamber pacing	16 (100)	8 (50)	8 (50)	–
Pacing programming				
DDD (R)	11 (69)	5 (31)	6 (38)	0.50
RV pacing avoidance algorithm	5 (31)	3 (18)	2 (13)	0.50
Pacing requirement				
Rate response	1 (6)	1 (6)	0 (0)	0.50
Base rate (bpm)	51 (± 6)	50 (± 5)	52 (± 8)	0.56
AP burden (%)	16 (± 24)	16 (± 22)	15 (± 28)	0.93
VP burden (%)	76 (± 40)	67 (± 46)	84 (± 34)	0.42
AF (%)	6 (± 25)	0 (± 0)	12 (± 35)	0.33
Resting heart rate (bpm)	57 (± 18)	60 (± 19)	54 (± 19)	0.58
Echocardiographic measurement				
LVEF (%)	53 (± 8)	54 (± 8)	51(± 8)	0.48
LVEDV (ml)	144 (± 40)	128 (± 46)	159 (± 29)	0.12
LVESV (ml)	69 (± 26)	59 (± 28)	78 (± 21)	0.14
GLS (%)	− 15 (± 4)	− 17 (± 3)	− 13 (± 3)	0.03*
Myocardial work				
GWE (%)	86 (± 9)	88 (± 5)	83 (± 12)	0.36
GWI (mmHg%)	1699 (± 457)	1819 (± 545)	1578 (± 342)	0.31
GCW (mmHg%)	2097 (± 586)	2304 (± 572)	1889 (± 557)	0.16
GWW (mmHg%)	327 (± 189)	306 (± 125)	348 (± 245)	0.68

Continuous data are presented as mean (± standard deviation) or median (interquartile range) and categorical data are presented as n (%). A *p-value ≤ 0.05 was considered significant

MI; myocardial infarction, PCI; percutaneous coronary intervention, CABG; coronary artery bypass graft, BNP; B-type natriuretic peptide, ACE inhibitors; angiotensin-converting enzyme inhibitor, β-blockers; beta-blockers, RV; right ventricular, AP; atrial pacing, VP; ventricular pacing, LVEF; left ventricular ejection fraction, LVESD; left ventricular end-systolic dysfunction, LVEDD; left ventricular end-diastolic diameter, GLS; global longitudinal strain, GWE; global work efficiency, GWI; global work index, GCW; global constructive work, GWW; global wasted work

**Table 2 (abstract ABS025):** Echocardiographic measurements at baseline and follow-up between CSP and RVP cohort

	CSP Group *n* *=* *8*			RVP Group *n* *=* *8*			p-value
	Baseline	Acute	Mid-term	Baseline	Acute	Mid-term	
LVEDV (ml)	128 (± 14)	101 (± 14)	119 (± 14)	159 (± 14)	127 (± 14)	137 (± 14)	0.60
LVESV (ml)	59 (± 9)	47 (± 7)	58 (± 8)	78 (± 9)	63 (± 7)	64 (± 8)	0.38
LVEF (%)	54 (± 3)	55 (± 2)	52 (± 3)	51 (± 3)	50 (± 2)	50 (± 3)	0.83
LVGLS (%)	− 17 (± 1)	− 14 (± 2)	− 14 (± 2)	− 13 (± 1)	− 13 (± 2)	− 13 (± 2)	0.39
LV MWI_S_ (mmHg%)	1411 (± 198)	1391 (± 199)	1446 (± 178)	1621 (± 198)	1210 (± 199)	954 (± 178)	0.04*
LV MWI_L_ (mmHg%)	1835 (± 263)	2013 (± 319)	1890 (± 243)	1283 (± 263)	1465 (± 319)	1788 (± 243)	0.44
LV MWI_S-L_ (mmHg%)	− 424 (± 270)	− 622 (± 306)	− 444 (± 176)	338 (± 270)	− 255 (± 306)	− 835 (± 176)	0.06

Continuous data are presented as mean (± standard error). A *p-value ≤ 0.05 was considered significant. LVEF, left ventricular ejection fraction; LVESD, left ventricular end-systolic dysfunction; LVEDD, left ventricular end-diastolic diameter; GLS, global longitudinal strain; LV MWIS, left ventricular myocardial work index of septal wall; LV MWIL, left ventricular myocardial work index of lateral wall; LV MWIs-L, left ventricular myocardial work index difference of septal and lateral wall

**Fig. 1 (abstract ABS025):**
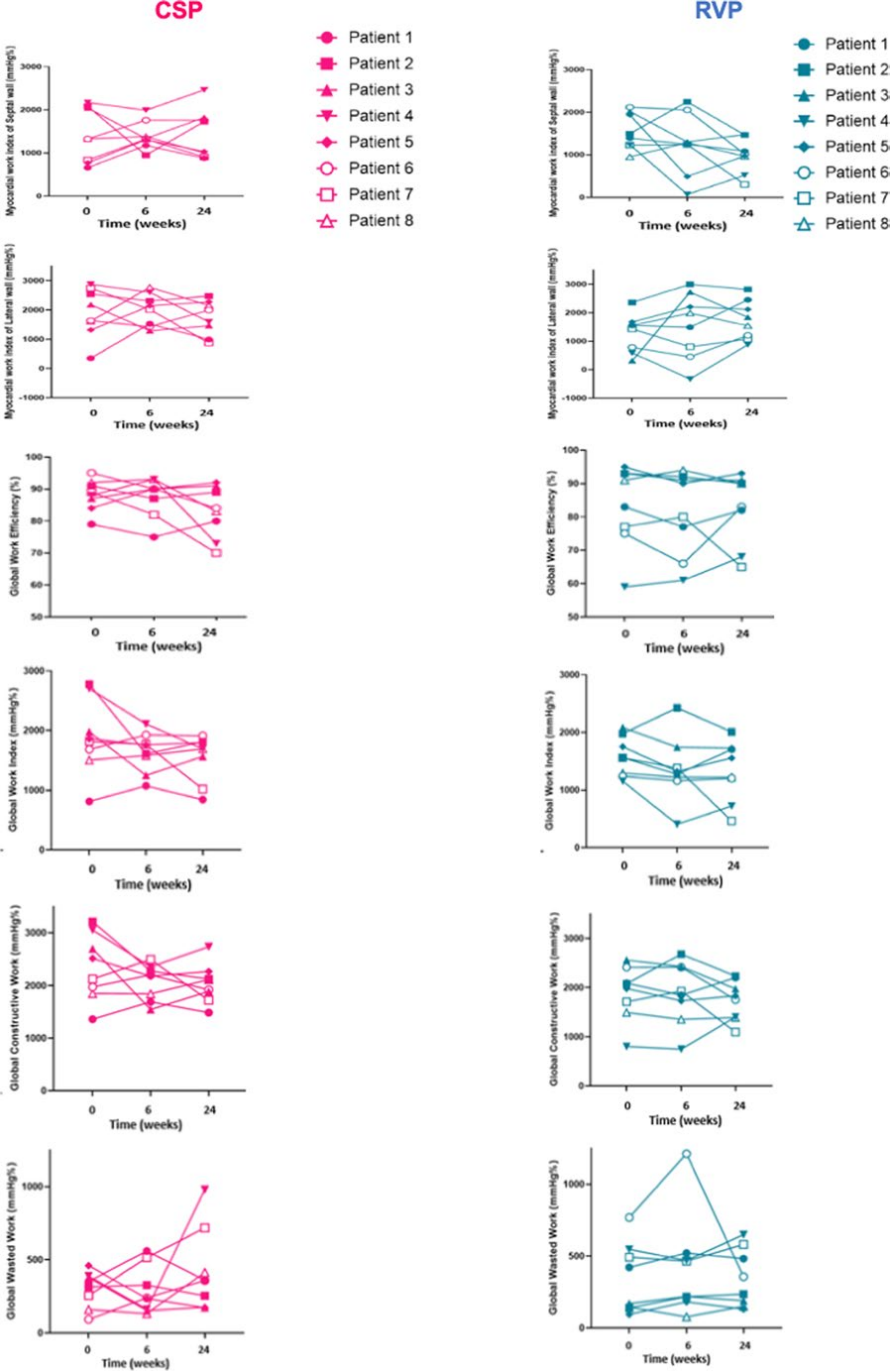
Myocardial Work indices change over time between CSP and RVP cohort during follow-up

## ABS026 A UK-wide Delphi consensus on the practical, consistent, and clinically appropriate approach to the reporting of the clinical suspicion of HFpEF using echocardiography

### Paton M. F.^1^, Barton C.^2^, Baruah R.^3^, Hartshorne-Evans N.^4^, Jenkins G.^5^, Potter A.^6^, Robinson S.^7^, Thakkar R.^8^, Zakeri R.^9^, Taylor C. J.^10^

#### ^1^Leeds Institute of Cardiovascular and Metabolic Medicine, University of Leeds, UK; ^2^Imperial College Healthcare NHS Trust, UK; ^3^AstraZeneca, London, UK; ^4^Pumping Marvellous, Preston, UK; ^5^NHS Wales, UK; ^6^Milton Keynes, UK; ^7^British Society of Echocardiography, UK; ^8^Primary Care Cardiovascular Society, UK; ^9^Kings College London, UK; ^10^Institute of Applied Health Research, University of Birmingham, UK

*Echo Research & Practice* 2025, **12(Suppl 1):**ABS026

**Introduction:** Heart failure with preserved ejection fraction (HFpEF) is a complex clinical syndrome where symptoms such as breathlessness and signs of heart failure (HF) occur in the setting of a normal left ventricular ejection fraction. Transthoracic echocardiography (TTE) is the first-line imaging modality for diagnosing HFpEF. Establishing a consensus on TTE reporting for HFpEF is expected to improve the diagnosis, management, and outcomes of HFpEF patients.

**Objectives:** To develop a clinically appropriate, and practical approach for reporting clinical suspicion of HFpEF using echocardiography.

**Methods:** Via a Delphi consensus method, a steering group of UK experts identified domains and formulated 34 statements relevant to the remit. Using a four-point *Likert* scale, a survey was disseminated with UK healthcare professionals. A consensus threshold of 75% agreement was defined.

**Results:** 135 UK specialists experienced in managing HF participated in the survey, including physiologists/clinical scientists (n = 43), specialist nurses (n = 35), cardiologists (n = 34), general practitioners (n = 12), pharmacists (n = 4), and others (n = 7). 30/34 (88%) statements achieved consensus within 7 domains; challenges in the system approach to HFpEF, enhancing referral for specialist review, confidence in utilising “*These findings may be consistent with HFpEF and should be considered in the context of symptoms and NT-proBNP*” in TTE reporting, identifying HFpEF and its underlying aetiology, HF awareness, training, and education, refining multidisciplinary team (MDT) roles, and optimising patient experience.

**Conclusion:** HFpEF diagnosis requires timely access to TTE coupled with specialist clinical assessment. Standardised practices for referral and TTE reporting which flags the possibility of HFpEF, alongside better integration of physiologists in the wider HF MDT, and improved education for generalists and specialists, may help eliminate diagnostic delays and geographical disparities.

## ABS027: Echocardiographic assessment of right ventricular structure and function in patients with arrhythmogenic cardiomyopathy: a systematic review with meta-analysis

### Gavin McClean^1^, Sara Delgado^1^, Abdulaziz Farooq^2^, Liam Ring^3^, David Oxborough^4^, Abbas Zaidi^5^

#### ^1^Royal Brompton & Harefield Hospitals, Part of Guy’s and St Thomas’ NHS Foundation Trust, London, UK; ^2^ASPETAR Qatar Orthopaedic and Sports Medicine Hospital, Doha, Qatar; ^3^West Suffolk Hospital NHS Trust, Bury St Edmunds, UK; ^4^Liverpool John Moore’s University, Liverpool, UK; ^5^University Hospital of Wales, Cardiff, UK

*Echo Research & Practice* 2025, **12(Suppl 1):**ABS027

**Background:** Transthoracic echocardiographic (TTE) plays an important role in imaging criteria for arrhythmogenic cardiomyopathy (ACM) diagnosis in combination with genetics/family history, and electrocardiographic findings. Diagnosis of right ventricular (RV) involvement by Task Force Criteria 2010 and the Padua Criteria necessitates subjective identification of RV regional wall motion abnormalities (RVWMAs), which can be challenging. Novel measures such as RV-free wall longitudinal strain (RVFWLS) in support of current criteria may improve ACM detection.

**Aim:** Establish the prevalence of RWMAs and the magnitude of RV structural and functional (including RVFWLS_ε_ and segmental RVFW_ε_) remodelling, in ACM patients compared to matched healthy participants.

**Design:** Systematic review with meta-analysis.

**Data sources:** Five electronic databases were searched using three concepts: 1) ACM 2) TTE 3) RV structure and function. Supplemented with forward citation tracking and ePublication lists of key journals.

**Selection Criteria and Risk of Bias:** (1) ACM patients aged ≥ 18 years (2) TTE by international standards (3) Original research articles published in English applied by two independent reviewers, with a third reviewer available for arbitration. Risk of bias was assessed independently by two reviewers using the Quality Assessment Tool for Quantitative Studies.

**Results:** Two thousand two hundred and thirty (2230) ACM patients and 763 healthy participants were included for qualitative and quantitative synthesis (43 articles; Fig. 1). RVOT_PLAX_ (p ≤ 0.001), RVOT_1_ (p ≤ 0.001), and RVD_1_ (p ≤ 0.01) were significantly larger in ACM patients vs. healthy participants (Table 1). RVWMAs were observed in 78% (95% CI 65–88) of ACM patients. RVFAC (p ≤ 0.001), RVFWLS_ε_ (p ≤ 0.001), RVFW Basalε (p ≤ 0.001), Mid-wallε (p ≤ 0.01), and Apicalε (p ≤ 0.001) were significantly lower in ACM patients vs. healthy participants (Fig. 2, Table 1). Sensitivity analysis including only case–control articles demonstrated no change in statistical significance across all parameters.

**Conclusion:** Inclusion of a RVWMA to detect morpho-functional abnormalities as per ACM diagnostic criteria may miss up to 22% of cases by TTE alone. Alternative measures of RH structure and function (including RVFWLS_ε_ and segmental RVFW_ε_) are significantly different between ACM patients and healthy participants and may provide quantitative detection of RVWMAs; thereby increasing diagnostic yield. Forthcoming TTE guidelines for ACM diagnosis should consider these data.

**Registration:** PROSPERO CRD42024541939.

**Fig. 1 (abstract ABS027):**
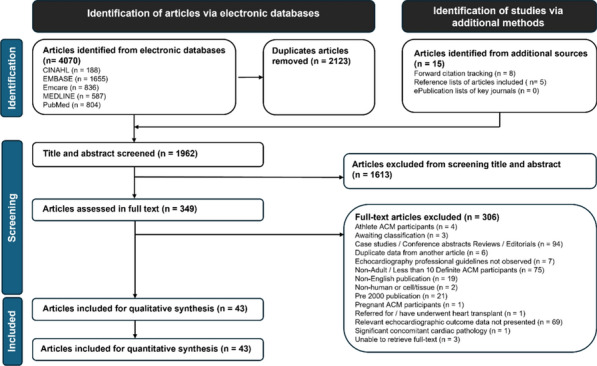
PRISMA flow chart of screened, included and excluded studies. PRISMA, Preferred Reporting Items for Systematic Reviews and Meta-Analyses

**Fig. 2 (abstract ABS027):**
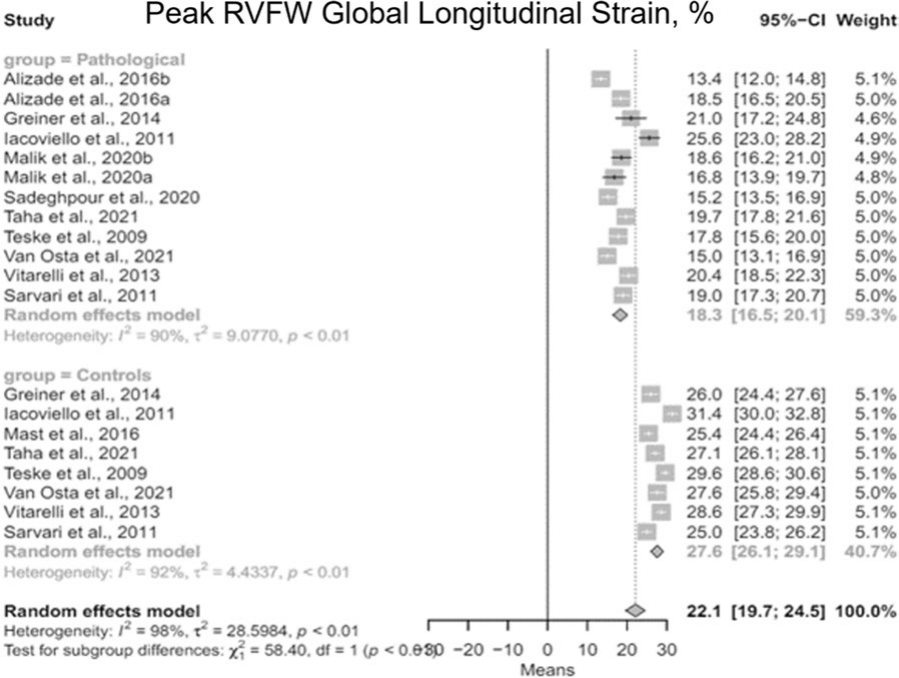
Forest plot of peak Right Ventricle Free Wall Global longitudinal strain (RVFWLS_ε_) in arrhythmogenic cardiomyopathy vs healthy participants

**Table 1 (abstract ABS027):** Demographics and right ventricle echocardiographic patterns of ACM and healthy participants

Parameter	ACM	Healthy participants	% Diff
Demographics			
Age, years	42.2 (40.5 to 43.9) [48, 2216] {94%}	39.4 (35.7 to 43.1) [22, 763] {98%}	7
BSA, m^2^	1.87 (1.79 to 1.95) [14,798] {95%}	1.87 (1.83 to 1.92) [8, 273] {67%}	0
Males, %	64.5 (61.5–65.6) [49, 2230] {53%}	58.3 (54.6–61.2) [22, 763] {95%}	10
RV size as per current criteria of ACM diagnosis			
RVOT_PLAX_, mm	36.5 (35.0 to 38.1)* [28, 1450] {97%}	27.9 (26.3 to 29.5) [8, 269] {92%}	24
RVOT_1,_ mm	36.0 (34.6 to 37.4)* [26, 1024] {99%}	29.0 (27.8 to 30.3) [9, 288] {80%}	19
RV size using parameters in extension to current criteria of ACM diagnosis			
RVEDA, cm^2^	27.8 (25.3 to 30.4) [15, 703] {96%}		
RVD_1_, mm	41.5 (38.1 to 44.8)^†^ [12, 337] {96%}	33.8 [29.5 to 38.1] [4, 136] {97%}	19
RVD_2_, mm	41.2 (35.4 to 47.0) [6,189] {92%}		
RVD_3_, mm	81.6 (77.5 to 85.6) [6,189] {88%}		
RV systolic function as per current criteria of ACM diagnosis			
RVWMA, %	78 (65.2 to 88)[14, 651] {89%}		
RVFAC, %	33.9 (32.1 to 35.6)* [31, 1306] {95%}	43.8 [41.2 to 46.4] [12, 459] {98%}	− 29
RV systolic function using parameters in extension to current criteria of ACM diagnosis			
RVFWLS ε, %	− 18.3 (− 16.5 to − 20.1)* [12, 314] {90%}	− 27.6 (− 26.1 to − 29.1) [8, 292] {92%}	− 51
RVFW Basal ε, %	− 16.8 (− 11.1 to − 22.5)* {4, 100} {92%}	− 27.7 (− 23.9 to − 31.5) {4, 127} {97%}	− 65
RVFW Mid-wall ε, %	− 19.5 (− 14.0 to − 24.9)^†^ [4, 100] {95%}	− 28.7 (− 24.6 to − 32.8) [4, 127] {97%}	− 47
RVFW Apical ε, %	− 22.5 (− 19.3 to − 25.8)* [3,83] {70%}	− 29.7 [− 27.2 to − 32.3] [3,105] {89%}	− 32

Data are mean (95% CI) [number of studies; number of participants] {heterogeneity}

*p ≤ 0.001, significantly greater in ACM than healthy participants

†p ≤ 0.01, significantly greater in ACM than healthy participants

ACM; arrhythmogenic cardiomyopathy Cm, centimetres; Diff, difference; m; meters; mm, millimetres; RV, Right ventricle; RVOT_PLAX_, RV outflow tract proximal dimension from a parasternal long axis orientation; RVOT_1_, RVOT proximal dimension from a short axis orientation; RVEDA, RV end-diastolic area; RVWMA, RV wall motion abnormalities (akinesia, dyskinesia, or aneurysmal); RVFAC, RV fractional area change; RVFWLS, RV Peak free wall global longitudinal strain

## ABS028 Can global longitudinal left ventricular strain aid detection of arrhythmogenic cardiomyopathy? A systematic review with meta-analysis

### Gavin McClean^1^, Sara Delgado^1^, Abdulaziz Farooq^2^, Liam Ring^3^, David Oxborough^4^, Abbas Zaidi^5^

#### ^1^Royal Brompton & Harefield Hospitals, Part of Guy’s and St Thomas’ NHS Foundation Trust, London, UK; ASPETAR Qatar Orthopaedic and Sports Medicine Hospital, Doha, Qatar; ^2^West Suffolk Hospital NHS Trust, Bury St Edmunds, UK; ^3^Liverpool John Moore’s University, Liverpool, UK; ^4^University Hospital of Wales, Cardiff, UK

*Echo Research & Practice* 2025, **12(Suppl 1):**ABS028

**Background:** Arrhythmogenic cardiomyopathy (ACM) is now recognised to affect not only the right ventricle, but also the left ventricle (LV), with biventricular and isolated LV disease seen in 70% and 17%, respectively upon autopsy. While the Padua criteria for imaging abnormalities includes LV diagnostic criteria, our understanding of LV involvement by transthoracic echocardiography (TTE) is limited.

**Aim:** Establish the presence of LV regional wall motion abnormalities (RWMAs) and the magnitude of LV structural and functional (including global longitudinal strain [GLS]) remodelling in ACM patients vs. matched healthy participants.

**Design:** Systematic review with meta-analysis.

**Data sources:** Five electronic databases using three concepts: (1) ACM (2) TTE (3) LV structure and function; supplemented with forward citation tracking and ePublication lists of key journals.

**Selection and Risk of Bias:** (1) ACM patients aged ≥ 18 years (2) TTE by international standards (3) Original research articles published in English applied by two independent reviewers, with a third reviewer available for arbitration. Risk of bias was assessed independently by two reviewers using the Quality Assessment Tool for Quantitative Studies.

**Results:** Two thousand, nine hundred and seventy-three (2973) ACM patients and 582 healthy participants were included for qualitative and quantitative synthesis (46 articles; Fig. 1). Visually detected LVRWMAs were observed in 37.9% (95% CI 29.5–47.1) of ACM patients. LVEF (57.4 [56.2–58.6] vs. 61.6 [60.5–62.7]; p ≤ 0.001) and GLS (− 17.6 [− 16.8 to − 18.3] vs. − 20.9 [− 20.0 to − 21.8]; p ≤ 0.001) were significantly reduced in ACM patients vs. healthy participants (Table 1; Fig. 2). Sensitivity analysis including only case–control articles demonstrated E/e’ to be significantly increased in ACM patients vs. healthy participants (10.4 [6.5–14.3] vs. 7.4 [4.6–10.2]; p ≤ 0.05).

**Conclusion:** The observation of LVRWMAs in ACM patients underlines the bi-ventricular pathophysiology of ACM. While LVEF was significantly lower, and E/e’ significantly higher in ACM patients, these parameters remained within normal reference limits. Therefore, in insolation, they’re unlikely to be clinically useful for detecting mild LV involvement in ACM. GLS, however, was significantly lower and fell into the borderline-low range in ACM patients; thus it may be a more useful clinical arbiter than E/e’ or LVEF. Future TTE criteria for diagnosing ACM should consider incorporating these novel findings.

**Registration:** PROSPERO CRD42024541939.

**Fig. 1 (abstract ABS028):**
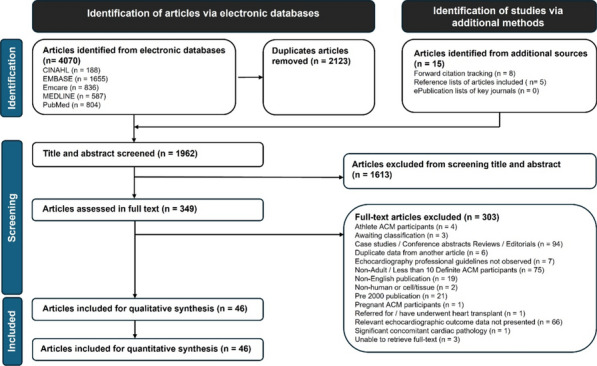
PRISMA flow chart of screened, included and excluded studies. PRISMA, Preferred Reporting Items for Systematic Reviews and Meta-Analyses

**Fig. 2 (abstract ABS028):**
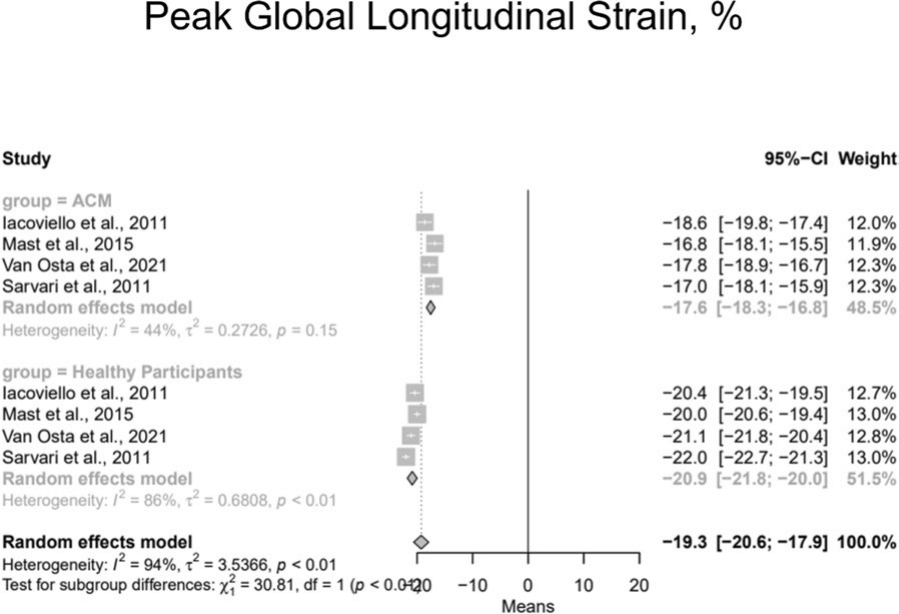
Forest plot of peak LV Global longitudinal strain in arrhythmogenic cardiomyopathy vs healthy participants

**Table 1 (abstract ABS028):** Demographics and left ventricle echocardiographic patterns of ACM and healthy participants

Parameter	ACM	Healthy participants	% Diff
Demographics			
Age, years	42.0 (40.4 to 43.6) [46, 2976] {90%}	40.1 (35.6 to 44.5) [16, 561] {95%}	5
BSA, m^2^	1.87 (1.78 to 1.96) [12, 756] {95%}	1.88 (1.83 to 1.93) [6, 208] {72%}	− 1
Males, %	63.0 (59.7 to 66.2) [42, 2973] {63%}	60.1 (54.2 to 65.8) [17, 582] {55%}	5
LV size and function			
LVIDd, mm	48.9 (48.4 to 49.3) [16, 1425] {36%}	50.0 (48.9 to 51.1) [3, 111] {72%}	− 2
LVEDV, ml	86.6 (70.0 to 103.2) [5, 189] {97%}		
LVEF, %	57.4 (56.2 to 58.6)* [34, 2740] {90%}	61.6 (60.5 to 62.7) [11, 449] {86%}	− 7
Visually detected RWMAs, %	37.9 (29.5 to 47.1) [6, 250] {63%}		
GLS, %	− 17.6 (− 16.8 to − 18.3)* [4, 130] {44%}	− 20.9 (− 20.0 to − 21.8) [4, 140] 86%}	− 19
E/A	1.4 (1.3 to 1.5) [6, 172] {82%}	1.5 (1.2 to 1.8) [4, 134] {95%}	− 7
Average E/e’	9.8 (7.6 to 11.9) [8, 190] {89%}	7.4 (4.6 to 10.2) [4, 134] {96%}	24

Data are mean (95% CI) [number of studies; number of participants] {heterogeneity}

*p ≤ 0.001, significantly greater in ACM than healthy participants

ACM; arrhythmogenic cardiomyopathy Diff, difference; m; meters; ml; millimetres; mm, millimetres

E/A; Transmitral E/A velocity; Average E/e’; Transmital E velocity/average (Septal and lateral) e’ by tissue Doppler imaging; GLS, Left ventricle peak Global Longitudinal Strain by Speckle Tracking Echocardiography; LVIDd, Left ventricle internal diameter during end-diastole; LVEDV, LV end-diastolic volume; LVEF, LV Ejection Fraction; RWMAs, LV regional wall motion abnormalities (Hypokinesia or akinesia of the LV myocardial segments)

## ABS030 Evaluating the appropriate use and clinical impact of pre-operative TTE prior to elective non-cardiac surgery

### James Tomlinson, Anil Gurung

#### Royal United Hospitals Bath, UK

*Echo Research & Practice* 2025, **12(Suppl 1):**ABS030

**Background/introduction:** Pre-operative transthoracic echocardiography (TTE) is commonly requested to assess left ventricular (LV) systolic function and valvular pathology prior to elective non-cardiac surgery (NCS). NICE recommend pre-operative TTE in patients with a heart murmur and cardiac symptoms, or if there are signs or symptoms of heart failure. Routine pre-operative TTE is not recommended prior to NCS.

**Purpose:** This audit aims to identify the following: number of patients referred for pre-operative TTE prior to elective NCS, appropriateness of referral by NICE guidance, mean wait times, proportion of scans which result in the identification of significant cardiac pathology and/or a change in clinical management.

**Methods:** Patients who underwent pre-operative TTE prior to elective NCS were identified in a 4 month period between January and April 2023. The indication for pre-operative TTE, mean wait times, findings and changes in clinical management were recorded.

## Results: 100 patients were identified to have undergone pre-operative TTE for elective NCS, 37% of which did not meet NICE guidance criteria for referral. There was an overall mean wait time of 42 days, with a mean wait time of 41 and 46 days for appropriate and inappropriate referrals respectively.

Severe and moderate valvular heart disease was demonstrated in 7 and 22 patients respectively. Severe (ejection fraction (EF) < 40%) and mid-range EF (41–49%) heart failure was identified in 4 and 5 patients respectively.

A change in clinical management occurred in 8 patients with 2 cases delayed until further cardiac optimisation and 6 cancelled with the patient deemed unfit for surgery.

**Conclusion:** Pre-operative TTE rarely results in a change of clinical management. Over a third of patients undergoing elective NCS are inappropriately referred for pre-operative TTE, associated with a mean wait time of 46 days. The inappropriate use of routine pre-operative TTE should, therefore be discouraged to avoid unnecessary resource use and delays to surgery which may potentially affect patient outcomes.

